# Dihydroisoxazole inhibitors of *Anopheles gambiae* seminal transglutaminase AgTG3

**DOI:** 10.1186/1475-2875-13-210

**Published:** 2014-06-02

**Authors:** Binh V Le, Cornelius Klöck, Alexandra Schatz, Jennifer B Nguyen, Evdoxia G Kakani, Flaminia Catteruccia, Chaitan Khosla, Richard HG Baxter

**Affiliations:** 1 Department of Chemistry, Yale University, P.O. Box 208107, New Haven, CT 06520-8107, USA; 2 Department of Chemistry, Stanford University, Stanford, CA 94305, USA; 3 Department of Molecular Biophysics and Biochemistry, Yale University, New Haven, CT 06511, USA; 4 Department of Immunology and Infectious Diseases, Harvard School of Public Health, Boston, MA 02115, USA; 5 Dipartimento di Medicina Sperimentale e Scienze Biochimiche, Università degli Studi di Perugia, 06123 Perugia, Italy

## Abstract

**Background:**

Current vector-based malaria control strategies are threatened by the rise of biochemical and behavioural resistance in mosquitoes. Researching mosquito traits of immunity and fertility is required to find potential targets for new vector control strategies. The seminal transglutaminase AgTG3 coagulates male *Anopheles gambiae* seminal fluids, forming a ‘mating plug’ that is required for male reproductive success. Inhibitors of AgTG3 can be useful both as chemical probes of *A. gambiae* reproductive biology and may further the development of new chemosterilants for mosquito population control.

**Methods:**

A targeted library of 3-bromo-4,5-dihydroxoisoxazole inhibitors were synthesized and screened for inhibition of AgTG3 in a fluorescent, plate-based assay. Positive hits were tested for *in vitro* activity using cross-linking and mass spectrometry, and *in vivo* efficacy in laboratory mating assays.

**Results:**

A targeted chemical library was screened for inhibition of AgTG3 in a fluorescent plate-based assay using its native substrate, plugin. Several inhibitors were identified with IC_50_ < 10 μM. Preliminary structure-activity relationships within the library support the stereo-specificity and preference for aromatic substituents in the chemical scaffold. Both inhibition of plugin cross-linking and covalent modification of the active site cysteine of AgTG3 were verified. Administration of an AgTG3 inhibitor to *A. gambiae* males by intrathoracic injection led to a 15% reduction in mating plug transfer in laboratory mating assays.

**Conclusions:**

A targeted screen has identified chemical inhibitors of *A. gambiae* transglutaminase 3 (AgTG3). The most potent inhibitors are known inhibitors of human transglutaminase 2, suggesting a common binding pose may exist within the active site of both enzymes. Future efforts to develop additional inhibitors will provide chemical tools to address important biological questions regarding the role of the *A. gambiae* mating plug. A second use for transglutaminase inhibitors exists for the study of haemolymph coagulation and immune responses to wound healing in insects.

## Background

Both historically and at present, vector control remains the most generally effective measure to prevent malaria transmission [1]. The two major control measures presently used are insecticide indoor residual spraying (IRS) and insecticide-treated bed nets (ITN). Both measures effectively target *Anopheles gambiae*, the principal malaria vector in sub-Saharan Africa, as it is both endophilic and endophagic. Yet IRS/ITN can be hindered by the selection of both biochemical and behavioural resistance in mosquitoes, driven by the same potent and rapid toxicity for which the insecticides are designed. Hence, while IRS/ITN will hopefully be successful in controlling and eliminating malaria, additional tools may be required to achieve the long-term goal of malaria eradication.

The sterile insect technique (SIT) is an alternative approach to insect control that has been successfully deployed against both agricultural pests and disease vectors [2-5], but never *Anopheles* mosquitoes. Classic SIT involves the mass release of sterile males, which for mosquitoes carries no disease risk because only females blood feed. Three methods of inducing sterility in males have been field tested on mosquitoes: gamma irradiation [6], chemosterilization [7-9], and genetic modification (GM) [10-13]. Despite its feasibility, the deployment of SIT for malaria control has been hindered by (i) logistical costs of gamma irradiation due to loss of fitness [14-16], (ii) perceived hazards associated with non-specific chemosterilants [17], and (iii) regulatory concerns with genetic modification [18-20].

Chemosterilization has generally delivered improved fitness compared to gamma irradiation [17], motivating a search for more specific and less toxic chemical agents to reduce the fertility of mosquitoes. To be applicable in the field a chemosterilant must meet the same efficiency and safety standards required from approved insecticides, it must not kill mosquitoes at the dose delivered nor change their mating behaviour, and if the compound is toxic or otherwise hazardous to the environment the released insect must be free of or contain a minimal residue of it [21]. The chitinase inhibitor luferunon, a benzoylurea derivative found in common flea control medications, has been successfully used as an edible bait for sterile control of fruit flies and related pests [22-24]. The DNA alkylating agent bisazir (P,P-bis(aziridin-1-yl)-*N*-methylphosphinothioic amide) was successfully used to chemosterilize *Anopheles albimanus* in field trials [25-28], but concerns over residual effects in non-target species [29] and potential health/environmental hazard limited operational deployment.

Hence, chemosterilants with improved specificity or a lower environmental hazard profile compared with bisazir could potentially advance the use of SIT in mosquitoes. The discovery of new chemosterilants would be enhanced by a better understanding of mosquito mating biology, including the function of numerous proteins of unknown function found in male seminal fluids. This goal would be advanced by identifying potential compounds targeting a specific enzyme within male seminal fluids that disrupts or inhibits the fertility of *A. gambiae*. The first target of interest identified was the male-specific protein AGAP009099, *A. gambiae* transglutaminase 3 (AgTG3).

**Figure 1:**
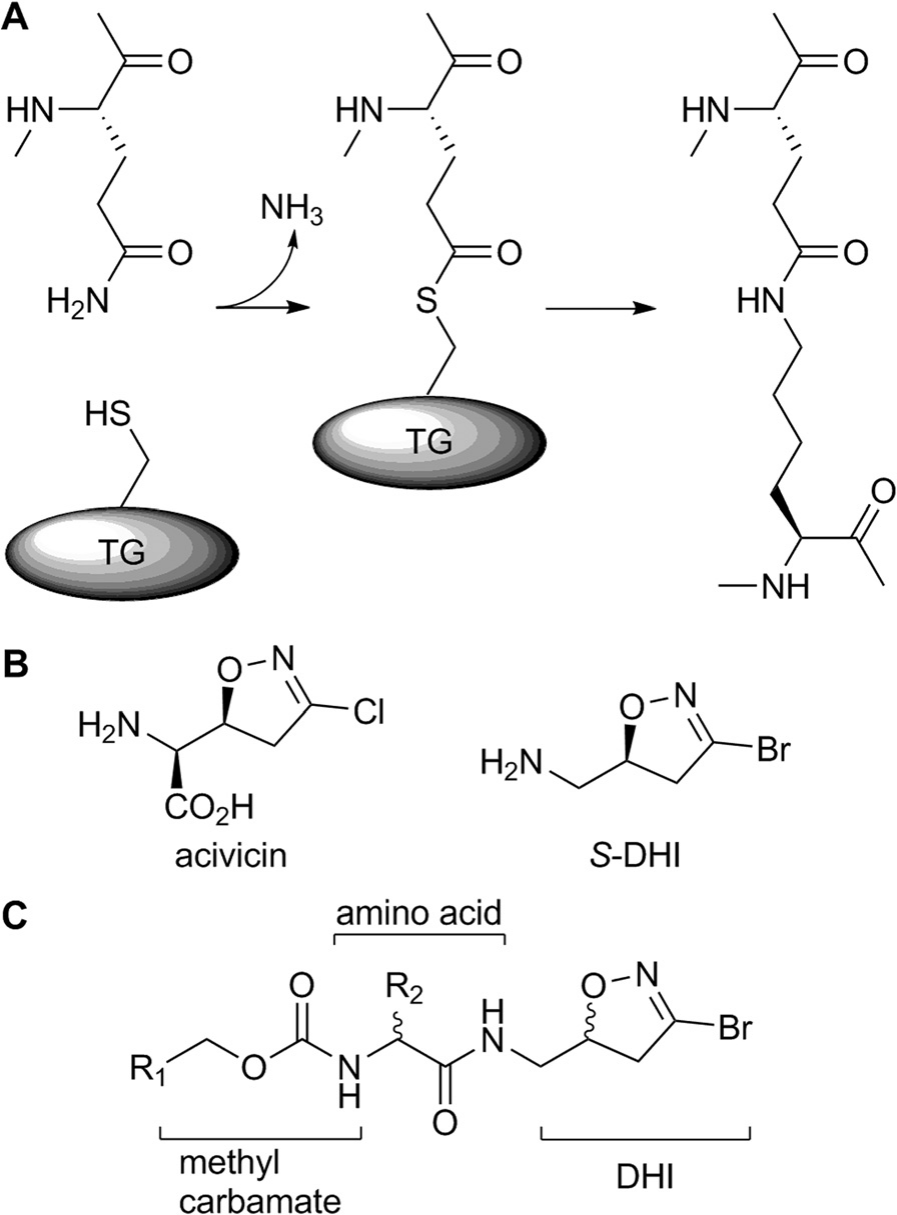
**Chemical mechanism of transglutaminase and DHI inhibitors. (A)** Mechanism of transglutaminase isopeptide bond formation. **(B)** Structure of acivicin, *S*-DHI-derived transglutaminase inhibitors.

Transglutaminases (TGs) catalyze the deamidation and transamidation of glutamine and the cross-linking of proteins by formation of ϵ-(γ-glutamyl)-lysine isopeptide bonds [30]. In mammals TGs are involved in blood clotting, formation of the epidermal barrier, cross-linking of the extracellular matrix, coagulation of seminal fluids, and contribute to the pathophysiology of cancer, inflammatory, autoimmune, and neurodegenerative diseases [31-33]. Of the eight active TGs in humans, tissue TG (hTG2) and blood clotting factor XIII (fXIIIa) have been the target of drug development for the treatment of disease. TGs are Ca2+-activated enzymes that rely on an active site cysteine to catalyze transamidation via a ‘ping-pong’ mechanism, in which the sulfhydryl attacks the glutamine group forming an acyl intermediate that is substituted by lysine (Figure 1A). The majority of TG inhibitors are tight, slow-binding inhibitors that react with the active site cysteine to irreversibly inhibit the enzyme [34].

*Drosophila melanogaster* has a single TG (DmTG) involved in cuticle morphogenesis and the coagulation of haemolymph in response to septic injury [35-37]. This TG is conserved in mosquitoes (*A. gambiae* AGAP009100, or AgTG1). *Culex* and *Anopheles* mosquitoes share a second TG gene (*A. gambiae* AGAP009098, or AgTG2), which has recently been implicated in the immune response to *Plasmodium falciparum*[38]. *Anopheles gambiae* has a third, male-specific TG, AgTG3, that coagulates male seminal fluids to form a ‘mating plug’ required for male reproductive success [39,40]. AgTG3 represents a specific, validated target for reducing male fertility in comparison to other broad-spectrum chemosterilants tested in mosquitoes. While there is some debate as to whether an inhibitor of AgTG3 would be an effective chemosterilant [40,41], an inhibitor of AgTG3 can be used as a chemical probe to analyse the role of the mating plug in the reproductive biology of *A. gambiae* and other seminal fluid proteins of unknown function.

The *in vitro* characterization of AgTG3 and cross-linking of its native substrate plugin have been previously reported [42]. Using a C-terminal fragment of plugin and fluorescein-cadaverine (FITC-CAD), a plate-based fluorescent assay was established and used to assay the activity of AgTG3 and inhibition by the halomethyl ketone iodoacetamide (IA), a potent but non-specific inhibitor of cysteine proteases and TGs. Besides iodoacetamide, a wide range of more specific halomethyl ketones. 3-halo-dihydroisoxazoles (DHIs), sulfonium methyl ketones (incl. thioimidazolium derivatives), epoxides, 1,2,4-thiadiazoles, diazomethyl ketones, maleimides and acryloyl amides (Michael acceptors) have been developed as TG inhibitors [34], Through synthesis, screening and SAR, TG inhibitors with IC_50_ < 100 nM and specificity for TG2 over fXIIIa have been developed [43,44].

DHI derivatives are based on the natural antibiotic acivicin (Figure 1B). Acivicin is non-toxic to *Drosophila* delivered orally *ad libitum* (10 μM in agar) and was active *in vivo* in a tumor model [45], while 3-bromoacivicin displayed no acute toxicity in mice at 50 mg/kg (intraperitoneal injection) and was active *in vivo* in a model of human African trypanosomiasis [46]. A series of DHI-based TG inhibitors synthesized and screened for activity against human TG2 [47-53] resulted in compounds that were tolerated at doses of 50 mg/kg *via* intraperitoneal injections in mice and were efficacious in blocking TG2 activity *in vivo*[51]. These studies suggest DHIs as a chemical class fulfill the criteria for compounds suitable as *in vivo* chemical probes or chemosterilants with an improved hazard profile compared to previously tested chemosterilants, such as bisazir.

Here, the assembly of a targeted chemical library of DHI and other TG inhibitors is reported, including previously characterized inhibitors of hTG2. Upon screening the library for inhibition of AgTG3 in the previously described FITC-CAD assay, several compounds were identified as potential inhibitors. These results were supported by dose–response experiments to determine the IC_50_ for a subset of structurally related compounds within the library. Inhibition of AgTG3 by the most active inhibitors was further validated *in vitro*. Injection of a DHI inhibitor into adult male *A. gambiae* provided a comparable effect to RNAi-mediated knock-down of AgTG3 in preventing transfer of the mating plug. These results are a proof-of-principle for the development of new classes of chemosterilants by molecular analysis of the reproductive biology of mosquitoes, and the use of TG inhibitors targeting both fertility and immunity in insects.

## Methods

### Library design and synthesis

The targeted library consisted of 92 compounds: 83 of the DHI chemotype and nine of alternate chemotypes. The DHI compounds were composed of previously reported hTG2 inhibitors [49-51,53], with additional unpublished derivatives (Additional file 1: Chemical Synthesis and Additional file 2: Table S1). The compound scaffold is an amino acid with an N-terminal protecting group and the DHI warhead attached to the C-terminus by an amide bond (Figure 1C). Alanine, aspartic and glutamic acid were present but the library was biased to l- and d- cyclic/aromatic residues and derivatives thereof. N-terminal protecting groups were biased towards aromatic methyl carbamates with several ethyl, *t*-butyl carbamates and two amides present. Initial compounds were racemic mixtures of (*S*/*R*)-DHI. As hTG2 is selectively inhibited by (*S*)-DHI, however, only 26 compounds in the library were racemates, another eight contained *R*-DHI and 49 were *S*-DHI. Besides DHI inhibitors, the library contained eight acylidene oxoindoles and the thienopyrimidinone LDN-27219 [54,55]. Acylidene oxoindoles are reversible, slow tight-binding inhibitors of hTG2 [55]; the thienopyrimidinones are allosteric inhibitors [54,56]. The binding site on TG2 is unknown for either chemotype.

**Figure 2:**
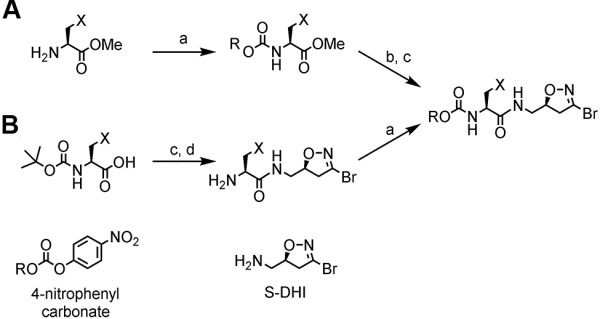
**Synthetic routes to generate DHI inhibitor library. (A)** Route for synthesis of previously reported DHI inhibitors. **(B)** Inverted route to utilize commercial BOC derivatives of amino acids. (a) 4-nitrophenyl carbonate, *N*-methylmorpholine, CH_2_Cl_2_, RT, overnight; (b) LiOH (1 eq.) MeOH/THF; (c) (*S*)-DHI, EDCI, HOBt, DMF; (d) TFA.

The DHI inhibitors were prepared according to the synthetic routes outlined in Figure 2. In one previously published sequence (route A), the amino acid methyl ester (free base or hydrochloride) is decorated with the desired carbamate substituent by reacting it with the respective 4-nitrophenyl carbonate building block. After saponification of the ester with aqueous lithium hydroxide, the (*S*)-DHI moiety can be introduced *via* standard amide coupling chemistry, furnishing the final inhibitor [49,50]. Recently, the reaction sequence was inverted (route B) to use the commercial abundance of BOC precursors [53]. Coupling of the BOC amino acid to the (*S*)-DHI moiety followed by BOC deprotection with TFA furnished an intermediate that could be directly coupled to the carbonate building block without further purification. The individual carbonate and (*S*)-DHI building blocks were prepared as previously published [47,50,57].

### AgTG3 FITC-CAD plate assay

Plugin-C (25 μg/well) was incubated in black 96-well Ni-coated plates (Pierce 15342) for 2 h at room temperature or overnight at 4°C. Plates were washed three times with 200 μL of TBS and loaded with 0.5 μg of AgTG3 in 50 μL TBS buffer. DHI inhibitors were added for 1 h to block the enzyme, followed by 50 μL of 2× assay buffer (2× TBS, 20 mM CaCl_2_, 100 μM FITC-CAD, 2 mM DTT). Wells were incubated for 60 min at room temperature, washed three times with 200 μL of TBS, and filled to a final volume of 100 μL for fluorescence measurements in a microplate reader (Biotek Synergy 2). The *z*-score was calculated based on the mean and standard deviation of three replicates of inhibitor compared to a DMSO control, $z_{i}=\left|{\bar{x}}_{\mathrm{DMSO}}-{\bar{x}}_{i}\right|/\left(\sigma_{\mathrm{DMSO}}+\sigma_{i}\right)$.

### AgTG3 plugin cross-linking assay

Recombinant AgTG3 was pre-incubated with 100 μM inhibitors overnight at room temperature. AgTG3, with and without pretreatment with inhibitors (1 μg), was added to plugin-C (20 μg) and 0.5 mM FITC-cadaverine (FITC-CAD) in 25 mM HEPES, pH 7.5, 10 mM CaCl_2_, 1 mM DTT. The reaction was incubated at room temperature for 2 h and quenched by heat denaturation of the proteins in the presence of Laemmli buffer. The extent of cross-linking was analysed by sodium dodecyl sulphate-polyacrylamide gel electrophoresis (SDS-PAGE). The gel was scanned using Alpha Imager 2200 software.

### LC-ESI mass spectrometry

Recombinant AgTG3 (20 μM) was dialyzed against HEPES buffer containing 100 mM NaCl, 10 mM CaCl_2_, and 5% glycerol. Inhibitors were added to a final concentration of 100 μM and incubated overnight. Samples were subsequently injected on a Waters ACQUITY Ultraperformance liquid chromatography (UPLC) system coupled to an ACQUITY UPLC Photodiode Array (PDA) λ detector. Electrospray ionization (ESI) mass spectrograms were collected in the positive ion mode and subsequently transformed for molecular weight determination using MASSLYNX software (Waters).

### Tandem MS/MS mass spectrometry

Samples prepared for ESI-MS (50 μL) were pretreated with 2 μL of 10 g/l trypsin (Sigma) for 1 h at 37°C. The reaction was stopped by formic acid, and the sample was submitted to the W.M. Keck Biotechnology Resource Laboratory (Yale University) for tandem MS/MS proteomics analysis. Results were further analysed using the online MassMatrix server.

### Cysteine protease inhibition assay

The activity of caspase-1 was determined by use of chromogenic substrate Ac-YVAD-pNA (Enzo Life Sciences, NY, USA). The assay was performed by adding 45 μL of 1 Unit caspase-1 (Abcam, MA, USA) and 5 μL of Ac-YVAD-pNA (final concentration, 5 μM) to 50 μL assay buffer (50 mM HEPES pH 7.4, 10 mM DTT, 1 mM EDTA, 0.1% CHAPS, 20% glycerol). Substrate cleavage after 1 h incubation at 37°C was measured by *p*-nitrophenyl acetate absorbance at 400 nm in a benchtop UV/Vis spectrophotometer (UV1800, Shimadzu, Kyoto, Japan).

The activity of recombinant tobacco etch virus (TEV) protease was determined by cleavage of a fusion protein containing a TEV cleavage site between GST and a 17 kDa fragment of human gp78. TEV and GST-gp78 were incubated at 37°C for 10 min at a substrate:enzyme mass ratio of 10:1. The reaction was stopped by adding 3× loading buffer (150 mM Tris–HCl pH 6.8, 300 mM DTT, 6% (w/v) SDS, 0.06% (w/v) bromophenol blue, 30% (v/v) glycerol). Samples were heated at 95°C for 3 min and separated by 4–20% gradient SDS-PAGE.

### *Anopheles gambiae* laboratory mating assays

*Anopheles gambiae* G3 strain mosquitoes from a laboratory colony were reared using standard conditions (26–28°C, 65–80% relative humidity, 12 h/12 h Light/Darkness (L:D) photoperiod). Mosquitoes were sexed as pupae and adult males and females were allowed to emerge in separate cages to ensure their status as virgins. Three-day-old virgin males were injected with 138 nl of 100 μM of inhibitor diluted in 5% DMSO, or with 138 nl of a 5% DMSO solution as a control. Mating experiments were performed four days after eclosion as described previously and couples were captured in copula [58]. RNAi experiments were performed as previously described [39]. Mated females were dissected within two hours of mating to determine the presence of the mating plug in their atrium.

## Results

### Primary screening results

**Figure 3:**
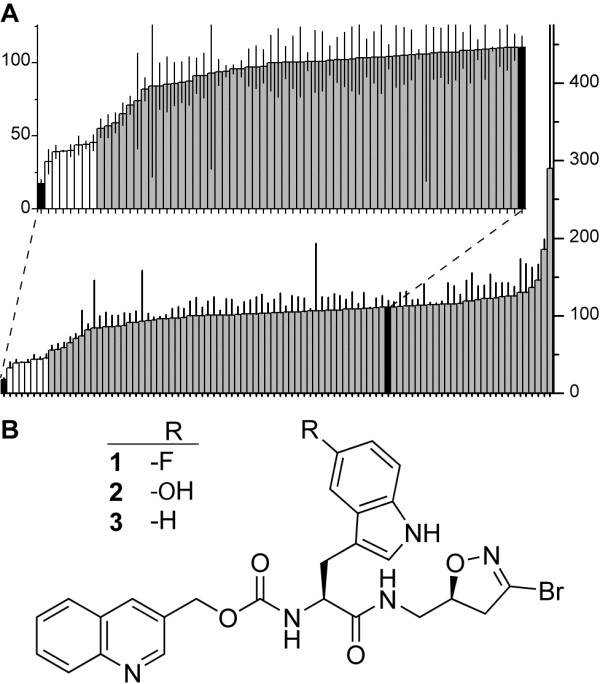
**Primary assay results for AgTG3 inhibitor screen. (A)** FITC-CAD/plugin-C cross-linking by AgTG3 incubated with 92 TG2 inhibitors, iodoacetamide and DMSO control (black), sorted by fluorescent intensity (arbitrary units). Hits shown in white. (inset) Expanded axes for compounds with *I* < DMSO, with error. **(B)** Common chemical structure of top three hits in primary assay.

The targeted chemical library was screened for inhibition of AgTG3 at 100 μM in a FITC-CAD plate assay (Figure 3A), performed in triplicate as previously described [50]. Hit-calling was based on >67% decrease in fluorescence compared to DMSO and *z* > 5. Seven compounds fulfilled these criteria in the primary assay. All were DHI inhibitors; neither acylidene oxoindoles nor the thienopyrimidinone LD-217912 showed any inhibition of AgTG3. Of the seven hits, the three most potent had a common structure (Figure 3B), quinolin-3-ylmethyl (*S*)-1-(((*S*)-3-bromo-4,5-dihydroisoxazol-5-yl)methylamino)-3-(5-R-1H-indol-3-yl)-1-oxopropan-2-yl-carbamate (Figure 3B), R = F (**1**), OH (**2**), H (**3**). Compounds **1**–**3** are also specific human TG2 inhibitors [50,51], which suggests a similar binding site exists for these inhibitors in the active site of AgTG3 and hTG2.

**Figure 4:**
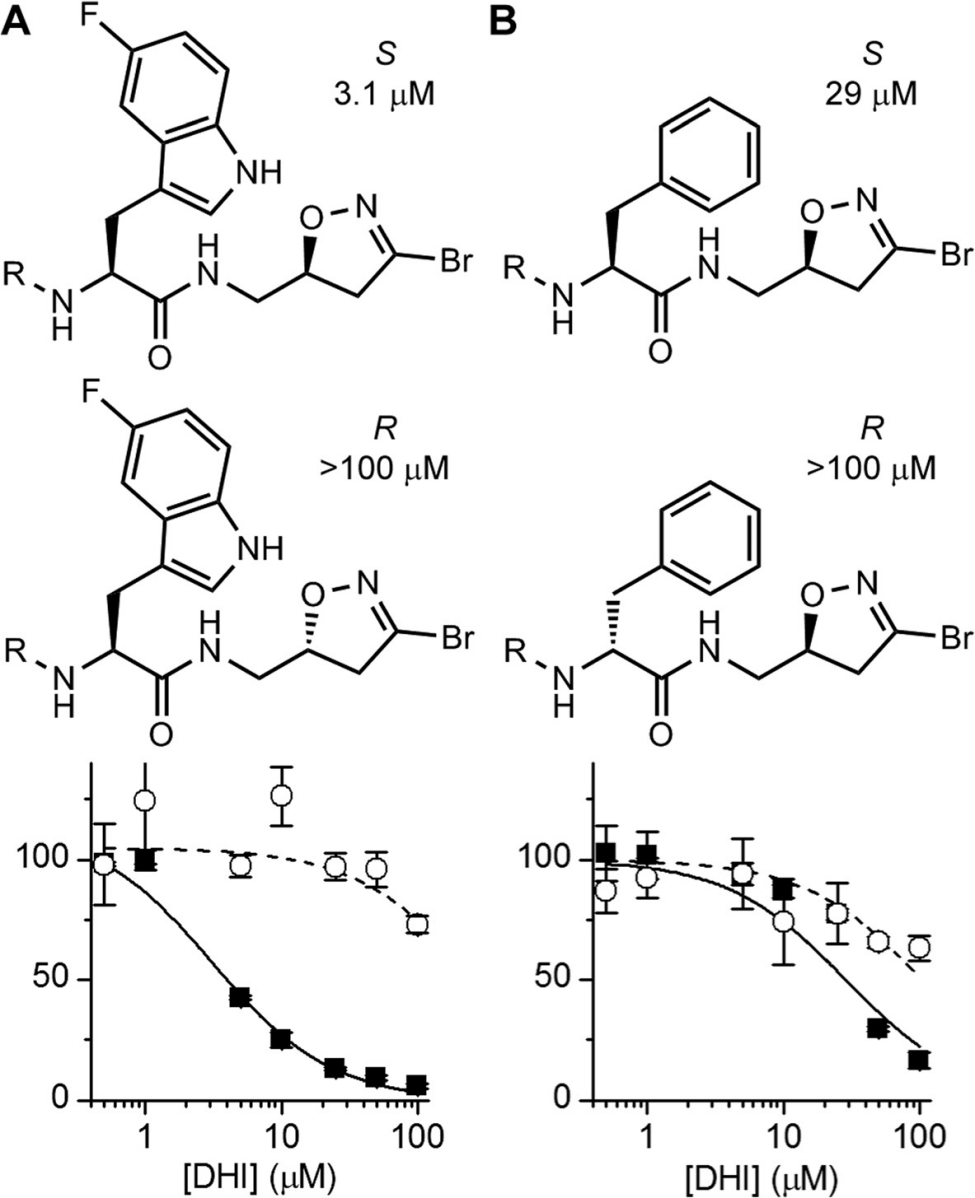
**Stereospecificity of AgTG3 inhibitors. (A)** Dose–response curves for 1 (S) and the corresponding (R)-DHI isomer (4). **(B)** Dose–response curves for the L-phenylalanine derivative of **1** (**5**) and the corresponding D-phenylalanine derivative (**6**). The structure of the carbamate substituent is quinolin-3-ylmethyl formate.

To verify the results of this preliminary assay, dose–response curves in the range of 0.5–100 μM were determined for 27 structurally related compounds within the targeted library (Additional file 3: Table S2 and Additional file 4: Table S3). The IC_50_ for **1**–**3** were 3.1 μM, 4.3 μM and 6.1 μM, respectively. Selected stereoisomers present in the targeted library were compared in equivalent dose–response assays. Inhibition of AgTG3 was specific for the (*S*)-DHI isomer of **1** (Figure 4A), the (*R*) isomer (**4**) displaying no activity (IC_50_ > 100 μM). Substitution of L-phenylalanine (**5**) for L-5-fluoro-tryptophan decreased activity (IC_50_ = 29 μM), but the corresponding D-phenylalanine (**6**) was inactive (Figure 4B). Hence AgTG3 inhibition is specific to (*S*)-DHI and L-amino acid isomers within the DHI inhibitor library, consistent with the known SAR for human TG2.

**Figure 5:**
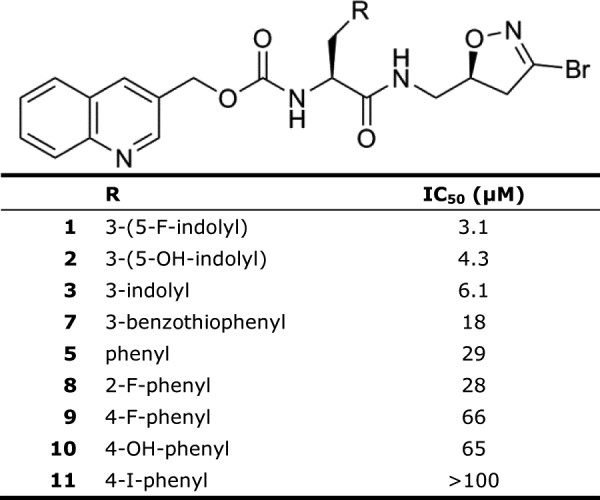
**IC**_**50 **_**of aromatic amino acyl side chains.**

The decreased activity of phenylalanine over tryptophan suggests the AgTG3 binding pocket has some selectivity for the amino acid directly conjugated to the DHI warhead. To examine this question dose–response curves of aromatic amino acid derivatives of the 3-quinolinylmethyl carbamate *S*-DHI inhibitor were compared (Figure 5). Compounds with indole or benzothiophene derivatives had lower IC_50_ values compared to phenyl or napthyl derivatives, implying a preference for a 5-membered *versus* 6-membered ring in the side chain. 2-fluorophenylalanine (**8**) had a similar activity (IC_50_ ~ 30 μM) compared to phenylalanine (**5**), but 4-fluorophenylalanine and tyrosine were less active (IC_50_ ~ 65 μM) and 4-iodophenylalanine was inactive at 100 μM.

**Figure 6:**
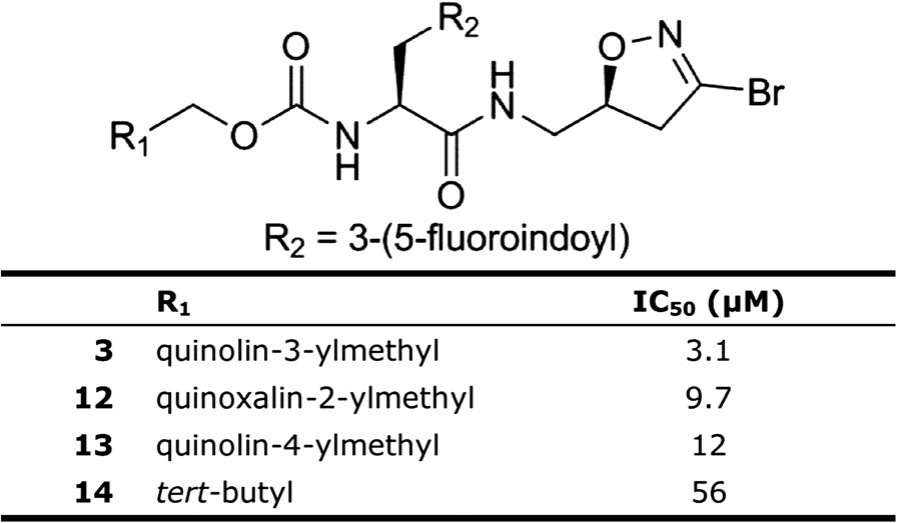
**IC**_**50 **_**of carbamate substituents.**

Due to the limited size of the compound library, only a small comparison set exists for different carbamate derivatives of compound **1** (Figure 6). Both quinoxalin-2-ylmethyl carbamate (**12**, IC_50_ 9.7 μM) and quinolin-4-ylmethyl carbamate (**13**, IC_50_ 12.2 μM) were AgTG3 inhibitors though slightly less active than compound **1**. Substitution of quinolin-3-ylmethyl with *tert*-butyl carbamate (**14**) led to a significantly less active compound (IC_50_ 56 μM), comparable to substitution of tyrosine as the amino acid (**10**) However, substituting the quinolin-3ylmethyl carbamate of **10** with either benzyl or fluorenylmethyl carbamate produced compounds with no activity at 100 μM.

### Secondary *in vitro* assays of AgTG3 inhibitors

**Figure 7:**
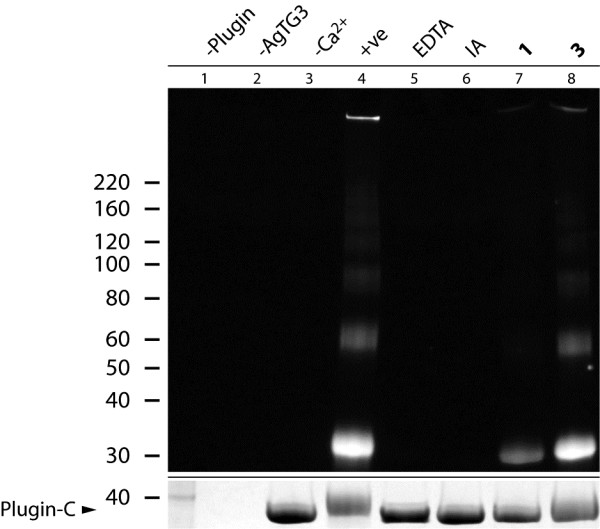
**Inhibition of plugin cross-linking by DHI inhibitors.** Fluorescent image of SDS-PAGE for FITC-cadaverine labeling and cross-linking of plugin by AgTG3. Lanes (1–4): (1) AgTG3, (2) Plugin-C, (3) AgTG3 and plugin-C, (4) AgTG3, plugin-C, and 5 mM CaCl_2_. Lanes (5–8): AgTG3, plugin-C, 5 mM CaCl_2_ and (5) 10 mM EDTA, (6) 50 mM IA, (7) 100 μM 1, (8) 100 μM 3. (below) Coomassie stain indicating relative levels of monomeric Plugin-C.

Additional assays were performed to examine whether compounds **1** and **3** would inhibit the cross-linking of plugin-C by AgTG3 in solution, with FITC-CAD for fluorescent detection of plugin-C. Results were assessed by SDS/PAGE and UV illumination to detect FITC-CAD (Figure 7). No fluorescence was observed for AgTG3 and plugin in the absence of calcium (lanes 1–3) since TGs are Ca^2+^-dependent enzymes. In the presence of enzyme, substrate and calcium a ladder of fluorescent bands of increasing molecular weight (MW) is observed (lane 4), corresponding to cross-linked oligomers of plugin also labeled by FITC-CAD. Cross-linking is inhibited by both EDTA (lane 5), which chelates calcium, and IA (lane 6), which blocks the active site cysteine. Compound **1** (lane 7) and, to a lesser extent, **3** (lane 8) inhibited the cross-linking of plugin as evidenced by reduced intensity of high MW bands. The fact that bands were observed at all, however, shows that inhibition was not as complete as for EDTA or iodoacetamide. However, the levels of AgTG3 in solution assays are higher in the cross-linking assay than the primary screen. The levels of plugin-C were also higher, which competes with slow-binding irreversible inhibitors such as **1** and **3** for the active site of AgTG3.

**Figure 8:**
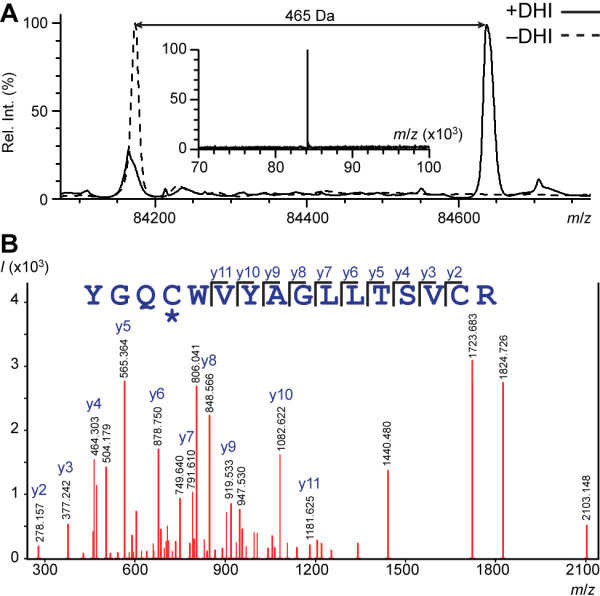
**Covalent modification of AgTG3 by DHI inhibitors. (A)** ESI-MS mass spectrum of AgTG3 incubated with 3 (solid line) vs. unmodified (dashed line). Inset: expanded mass spectrum for unmodified AgTG3. **(B)** Ion fragmentation spectrum AgTG3 tryptic peptide containing active site cysteine modified by 3 (*m*/*z* 2287.87).

DHI inhibitors form a thioether bond with the TG active site cysteine [34], a modification that should be detectable by mass spectrometry. Since **3** appeared less effective in a solution cross-linking assay than the primary assay, it is necessary to confirm covalent modification of the active site cysteine. AgTG3 was treated with 100 μM **3** overnight and the intact protein subsequently analyzed by ESI-MS (Figure 8A). The parent mass for unmodified AgTG3 was detected at *m*/*z* = 84173 Da. Following overnight treatment with **3**, the parent mass was significantly reduced and a new mass detected at *m*/*z* = 84638 Da. The +465 *m*/*z* shift strongly suggested specific modification of AgTG3. The expected *m*/*z* shift for reaction with **3** is +470, however, +5 amu than what was observed. To confirm the mass shift corresponds to modification of the active site cysteine with **3**, treated AgTG3 was subjected to tryptic digestion and LC-MS/MS analysis. A +469 amu mass shift of the tryptic peptide containing the AgTG3 active site cysteine (Cys 323) was detected, with *y* ion fragmentation confirming specific modification of Cys 323 (Figure 8B). No other modified cysteines were detected in the protein. Taken together, the secondary *in vitro* assays of cross-linking and covalent modification confirm **1**–**3** as active site inhibitors of AgTG3.

### Lack of inhibition of cysteine proteases

**Figure 9:**
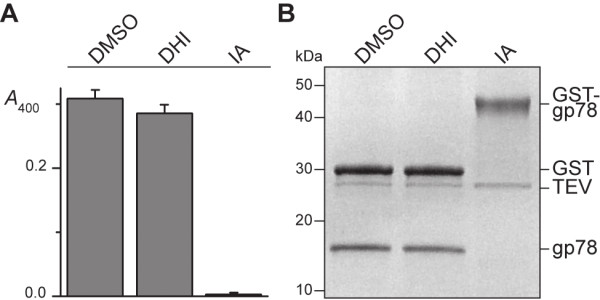
**DHI inhibitor inactive for two cysteine proteases.** Cleavage of **(A)** Ac-YVAD-pNA by caspase-I, or **(B)** GST-gp78 fusion protein by TEV, in the presence of TG inhibitors: (i) –ve control (DMSO), (ii) 3 (DHI), (iii) iodoacetamide (IA).

Transglutaminases and cysteine proteases have a similar catalytic mechanism involving an active site cysteine and formation of a thioacyl intermediate. Hence, inhibitors **1**–**3** may not be selective for AgTG3 but also inhibit cysteine proteases. To test this hypothesis, the inhibition of two cysteine proteases, human caspase-1 and recombinant tobacco etch virus (TEV) protease by **3** at 100 μM were tested using a chromogenic and SDS-PAGE assay, respectively (Figure 9). In both cases, the DHI inhibitor showed no inhibition of the protease compared to a DMSO control, while IA was a potent inhibitor of the enzyme. Neither human caspase-1 nor TEV are endogenous proteins in *A. gambiae*, which may well contain other endogenous targets of **1**–**3**, not least the two other transglutaminases in the *A. gambiae* genome. Nevertheless, the data suggests that **1**–**3** are not broad-spectrum inhibitors of cysteine-containing enzymes.

### Inhibition of mating plug formation

Finally, *A. gambiae* laboratory mating assays were performed to test if the DHI inhibitors identified in the pilot screen could inhibit male transfer of the mating plug. Compound **3,** 100 μM in DMSO, was administered to male mosquitoes by intrathoracic injection. Injected males were mated with virgin females, and their ability to transfer a mating plug to females was assessed (Table 1). In three independent experiments, 11 out of 75 females (15%) mated to DHI-injected males failed to receive a mating plug, compared to 2 out of 59 (3%) females mated to males injected with DMSO. The fraction of DHI-treated males that failed to transfer a mating plug is comparable to that previously observed for RNAi-mediated knock-down of AgTG3 [39]. In simultaneous experiments using ds*AgTG3* and ds*LacZ*, 5 of 77 (7%) females mated to ds*AgTG3*-injected males failed to receive a plug, compared to only 2 of 119 (2%) females mated to ds*LacZ*-injected mosquitoes. Thus, injection of males with AgTG3 inhibitor **3** is at least as effective as injection of dsRNA to inhibit mating plug formation in *A. gambiae* males.

**Table 1:** ***Anopheles gambiae *** **laboratory mating assay**

	3***	**DMSO**	***dsAgTG3*** *	***dsLacZ***
*N*	75	59	77	117
Mating plug transferred	64	57	72	119
No plug transferred	11	2	5	2

*p < 0.05 for a cumulative binomial test, *p*(no plug) = 0.034.

***p < 0.001 for a cumulative binomial test, *p*(no plug) = 0.034.

## Discussion

**Figure 10:**
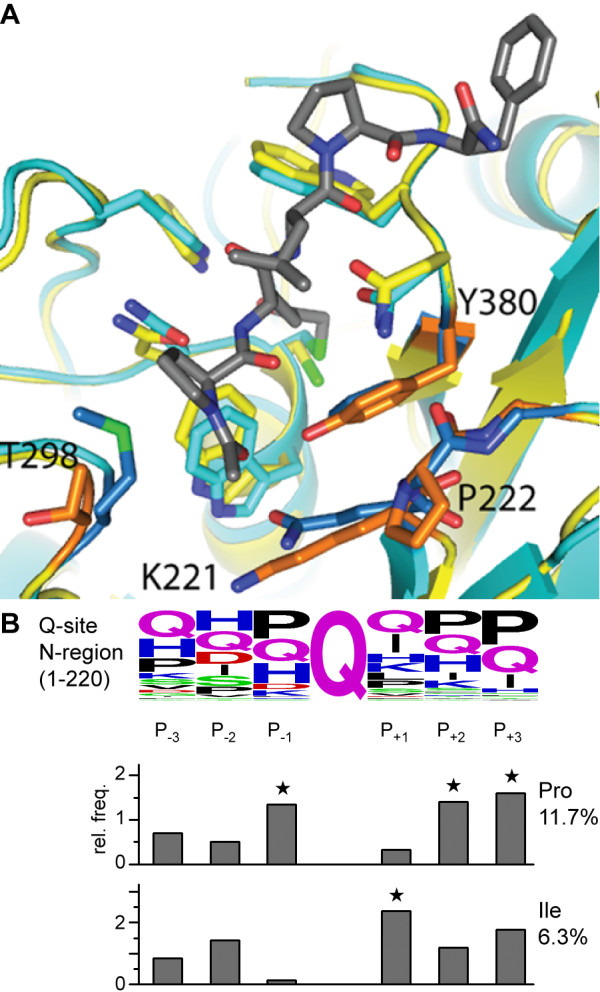
**Model of the AgTG3 active site. (A)** Homology model of AgTG3 (yellow) superimposed with TG2-inhibitor complex 2Q3Z (cyan). Non-conserved AgTG3 residues (K221, P222, T298, Y380) are shown (orange) in comparison to TG2 (blue) in the peptide-binding groove. **(B)** Proline is highly enriched at the P_-1_ relative to Gln in the N-terminal region of Plugin, justifying the bias towards cyclic amino acid variants in the initial library.

A number of DHI inhibitors of the *A. gambiae* male seminal transglutaminase AgTG3 have been identified through a targeted pilot screen. The best inhibitors identified (**1**–**3**) are also potent inhibitors of human TG2. This suggests that the inhibitor may adopt a similar conformation in the active site in the enzyme. Indeed, while AgTG3 is only ~30% identical in sequence to mammalian transglutaminases the homology within the active site is significantly higher. No crystal structure of a TG in complex with a DHI inhibitor is available, but the structure of TG2 in complex with a peptidomimetic inhibitor is known [59]. A homology model of AgTG3 based on the structure of the TG2-inhibitor complex illustrates the conservation of aromatic and Asn residues that surround the active site cysteine (Figure 10A), supporting the hypothesis of a common binding pose for the inhibitor.

Assuming that bound DHI adopts a conformation in which the Oγ is H-bonded to Trp 288 and Gln 322, the quinolinyl- and indole moieties of (**1**–**3**) could bind within the common peptide binding groove for both enzymes. There are some differences in amino acid residues within the groove that may present opportunities for improvement in the affinity and selectivity of AgTG3 inhibitors. Recent structures of fXIIIa in the active conformation provide a second starting point for modeling of the AgTG3-DHI active site [60]. Modeling combined with kinetic, SAR and structural studies may lead to more potent active site inhibitors of AgTG3. Future optimization could also include substitution of the DHI functional group with other cysteine-reactive functionalities [34].

No cyclic amino acid variants of the DHI inhibitor series displayed any inhibition of AgTG3 in the primary assay. A bias for cyclic amino acids in the targeted library was supported by the observation that plugin is enriched in Pro-Gln-Ile repeats within the N-terminal low complexity region (Figure 10B) [42]. The lack of activity for cyclic DHI inhibitors suggests that P_+1_ interactions (such as Ile) are important for the affinity of Plugin cross-linking sites for AgTG3, which is not reflected in the scaffold of the existing library. Likewise, neither the eight acylidene oxoindoles tested nor the thienopyrimidinone LDN-27219 inhibited AgTG3 at 100 μM, despite all having an IC_50_ < 10 μM for hTG2 [54-56], suggesting the (unknown) binding site on hTG2 for these compounds is not present in AgTG3. For instance, hTG2 has a binding site for GTP, which functions as negative allosteric regulator [61], but this site is not conserved in AgTG3 [42]. It remains plausible however, that allosteric inhibitors of AgTG3 exist, and present an alternative route toward a more selective AgTG3 inhibitors.

The observed *in vivo* efficacy of **3**, a 15% reduction in transfer of the mating plug, is low. Nevertheless, this result is significant as it represents the first report of efficacy for a putative chemosterilant specifically designed to inhibit a seminal fluid protein in a mosquito, indeed for any insect. The low efficacy observed may be due to rapid degradation of the compound in the haemolymph or limited accessibility of the compound to the male accessory glands (MAGs). Indeed, Cbz-tryptophan DHI inhibitors have been considered unsuitable candidates for human drugs due to metabolic instability [50]. Both the carbamate linkage and the amide bond between the amino acid core are susceptible to hydrolysis. Hence, the first goal for future synthetic development should be substitution or modification of these reactive bonds while simultaneously improving potency (IC_50_) and specificity (*k*_inact_/*K*_I_) of the inhibitor.

An important caveat to the laboratory mating assay is that the effect observed may not be a direct effect of AgTG3 inhibition but an off-target effect of the inhibitor. *Anopheles gambiae* has two other TGs that may also be inhibited by compounds **1**–**3**. The role of these TGs includes haemolymph coagulation and response to wounding. However, males injected with **3** showed no obvious ill effect from wounding, suggesting that if AgTG1 or AgTG2 are inhibited the effect is not systemic. Another concern is reactivity of the carbamate, especially considering the similarity of quinolinyl-3-ylmethyl carbamate to existing carbamate insecticides that target acetylcholinesterase (AChE). Hence, the second goal for future development of a mechanistic probe of AgTG3 is (i) develop assays for inhibition of known potential targets such as AgTG1 and AgTG2, and (ii) validate that the observed effect in laboratory mating access results from on-target activity, such as by isolating and analyzing the activity of endogenous AgTG3 from injected males, or synthesizing an identical but non-reactive inhibitor (i.e. lacking the halogen leaving group) as a control compound for *in vivo* assays.

A priority for future development is to establish well-defined criteria that an adequate chemical probe must meet, based on the broad requirements for a chemosterilant [21]. Molecular criteria for bioavailability of agrochemicals have been proposed based on analysis of commercially available agents and high-throughput screening [62], emphasizing low molecular weight (200–400 amu) and low polarity (logP = 3 ± 3) since topical application is the predominant route of access. For a laboratory probe molecule however, intrathoracic injection is a common procedure if a topical or orally available inhibitor cannot be obtained. Criteria for the potency, specificity and lifetime of the molecule *in vivo* must be empirically established based on the specific mating experiments, based on the principle that treated males must display equivalent mating fitness to untreated males yet a statistically significant defect in fertility.

While the role of AgTG3 is of specific interest to *A. gambiae*, the common role of insect TGs is haemolymph coagulation, cuticle morphogenesis and wound healing. DmTG knockdown has a pupal semi-lethal phenotype and abnormal morphology [35], and flies with reduced TG function show increased mortality after septic injury, suggesting an immune defect [36]. DmTG knockdown leads to a significant reduction in lifespan in *D. melanogaster* adults reared in normal, but not germ-free, conditions [37]. Interestingly, this result seems due to decreased immune tolerance to commensal bacteria rather than the suppression of immune responses to infection. The same function presumably exists for the conserved TG in mosquitoes (AgTG1). The role of the second TG shared by *Culex* and *Anopheles* is not clear, but AgTG2 was recently reported to be up-regulated by wounding and involved in injury-induced immune responses that are cross-reactive against human malaria [38]. Hence, a second potential role for TG inhibitors is as immunosuppressants that may act as slow-acting insecticide. Further study of, and development of inhibitors for, insect TGs should be pursued with the goal of developing new strategies to target mosquito fertility and immunity, for the future control of vector-borne disease.

## Competing interests

The authors declare no competing financial interests.

## Authors’ contributions

BVL, AS and JBN, FC and RHGB designed experiments. CK designed and performed DHI synthesis. BVL, AS, JBN and EK performed experiments. All authors contributed to and commented on the manuscript.

## Supplementary Material

Additional file 1 Chemical Synthesis. Click here for file

Additional file 2: Table S1 TG inhibitor library structures. Click here for file

Additional file 3: Table S2 AgTG3 IC50 for 27 dihydroisoxazole inhibitors. Click here for file

Additional file 4: Table S3 Graphical comparison of AgTG3 IC50 for 27 dihydroisoxazole inhibitors. Click here for file
