# Supplementary material for: Dihydroisoxazole inhibitors of Anopheles gambiae seminal transglutaminase AgTG3
Source: Malar J. 2014 Jun 2;13:210. doi: 10.1186/1475-2875-13-210 (PMC4113009; doi:10.1186/1475-2875-13-210)

Chemical Synthesis of TG inhibitors

quinolin-3-ylmethyl ((S)-1-((((S)-3-bromo-4,5-dihydroisoxazol-5-yl)methyl)amino)-3-(1H-indol-3-yl)-1-oxopropan-2-yl)carbamate (**1**) and **quinolin-3-ylmethyl ((S)-1-((((S)-3-bromo-4,5-dihydroisoxazol-5-yl)methyl)amino)-3-(5-hydroxy-1H-indol-3-yl)-1-oxopropan-2-yl)carbamate** (**2**) were synthesized as reported elsewhere (Dafik & Khosla, 2011).

quinolin-3-ylmethyl ((S)-1-((((S)-3-bromo-4,5-dihydroisoxazol-5-yl)methyl)amino)-3-(5-fluoro-1H-indol-3-yl)-1-oxopropan-2-yl)carbamate (**3**), its diastereomer **quinolin-3-ylmethyl ((S)-1-((((R)-3-bromo-4,5-dihydroisoxazol-5-yl)methyl)amino)-3-(5-fluoro-1H-indol-3-yl)-1-oxopropan-2-yl)carbamate** (**4**) and **quinolin-3-ylmethyl ((S)-1-((((S)-3-bromo-4,5-dihydroisoxazol-5-yl)methyl)amino)-3-(4-hydroxyphenyl)-1-oxopropan-2-yl)carbamate** (**10**) were prepared as described previously (Watts, Siegel, & Khosla, 2006).

quinolin-3-ylmethyl ((S)-1-((((S)-3-bromo-4,5-dihydroisoxazol-5-yl)methyl)amino)-1-oxo-3-phenylpropan-2-yl)carbamate (**5**):
L-Phenylalanine ethyl ester hydrochloride (240 mg, 1.045 mmol) and 4-nitrophenyl (quinolin-3-ylmethyl) carbonate (400 mg, 1.233 mmol) were placed in a 25 mL round-bottomed flask and dissolved in DMF (Volume: 8 ml) to yield a yellow solution to which N-Methylmorpholine (0.172 ml, 1.567 mmol) was added in one aliquot. The mixture was stirred for 16 h and then diluted with 100 mL of ethyl acetate and 50 mL of DCM. The organic phase was washed saturated aqueous bicarbonate (3x 50 mL) and brine (1 x 50 mL) and dried over anhydrous sodium sulfate and potassium carbonate. The volatiles were removed under reduced pressure. The resulting yellow oil was purified by flash chromatography (1:1 EtOAc : Hexanes - 5:5:1 EtOAc : Hexanes : Ethanol) to afford (S)-methyl 3-phenyl-2-(((quinolin-3-ylmethoxy)carbonyl)amino)propanoate (190 mg, 0.521 mmol, 49.9 % yield). The methyl ester was dissolved in THF (2 ml). LiOH (1M) (0.521 ml, 0.521 mmol) was added in one aliqout, followed by few drops of methanol to dissolve the resulting suspension. The mixture was stirred for 3 h, acidified with HCl and extracted with ethyl acetate (3x 15 mL). The combined organic layers were dried over sodium sulfate and concentrated under reduced pressure to yield (S)-3-phenyl-2-(((quinolin-3-ylmethoxy)carbonyl)amino)propanoic acid (95 mg, 0.271 mmol, 52 % yield) as a white solid, which was directly dissolved in DMF (2 ml), and hydroxybenzotriazole (36.6 mg, 0.271 mmol), (S)-DHI (48.5 mg, 0.271 mmol) and EDCI (52.0 mg, 0.271 mmol) added. The mixture was stirred at room temperature for 18 h and then diluted with 40 mL of ethyl acetate. The organic phase was washed with water (4 x 10 mL) and then dried over sodium sulfate. The solvent was removed and the residual yellow oil was purified by flash chromatography (9 : 1 - EtOAc : Hexanes). quinolin-3-ylmethyl ((S)-1-((((S)-3-bromo-4,5-dihydroisoxazol-5-yl)methyl)amino)-1-oxo-3-phenylpropan-2-yl)carbamate quinolin-3-ylmethyl (43 mg, 0.084 mmol, 31.0 % yield) was obtained as a white solid. ^{1}H NMR (400 MHz, DMSO-d_6) δ 8.84 (d, *J* = 2.1 Hz, 1H), 8.39 (t, *J* = 5.9 Hz, 1H), 8.25 – 8.20 (m, 1H), 8.02 (d, *J* = 8.4 Hz, 1H), 7.94 (d, *J* = 8.3 Hz, 1H), 7.76 (ddd, *J* = 8.4, 6.9, 1.5 Hz, 1H), 7.69 – 7.58 (m, 2H), 7.31 – 7.10 (m, 5H), 5.23 – 5.10 (m, 2H), 4.69 (tdd, *J* = 12.4, 6.0, 4.4 Hz, 1H), 4.26 (ddd, *J* = 10.4, 8.8, 4.5 Hz, 1H), 3.47 –3.18 (m, 3H), 3.00 – 2.89 (m, 2H), 2.76 (dd, *J* = 13.6, 10.6 Hz, 1H). ^{13}C NMR (101 MHz, DMSO-d_6) δ 172.97, 156.42, 151.26, 147.76, 138.83, 138.64, 135.24, 130.71, 130.31, 129.82, 129.43, 128.72, 128.68, 127.88, 127.62, 126.96, 80.74, 63.95, 56.96, 44.11, 41.92, 38.33.HRMS (ESI-QTOF) m/z: calculated for $C_{24}H_{24}BrN_4O_4^+$ $[M+H]^+$ 511.09754; found 511.09725.

quinolin-3-ylmethyl ((R)-1-((((S)-3-bromo-4,5-dihydroisoxazol-5-yl)methyl)amino)-1-oxo-3-phenylpropan-2-yl)carbamate, TFA salt (**6**):
The title compound was prepared from D-phenylalanine methyl ester by the same method used to prepare compound (**5**) and was purified by preparative TLC and reverse-phase HPLC in in water/acetonitrile with trifluoroacetic acid. ^{1}H NMR (500 MHz, DMSO-d_6) δ 8.95 (d, *J* = 2.1 Hz, 1H), 8.48 – 8.40 (m, 1H), 8.37 (t, *J* = 6.0 Hz, 1H), 8.08 (d, *J* = 8.5 Hz, 1H), 8.06 – 7.97 (m, 1H), 7.86 (ddd, *J* = 8.4, 6.9, 1.4 Hz, 1H), 7.71 (ddd, *J* = 8.1, 6.9, 1.2 Hz, 1H), 7.67 (d, *J* = 8.5 Hz, 1H), 7.35 – 7.08 (m, 5H), 5.29 – 5.12 (m, 2H), 4.69 (ddt, *J* = 10.5, 7.7, 5.1 Hz, 1H), 4.23 (ddd, *J* = 10.5, 8.4, 4.4 Hz, 1H), 3.42 – 3.22 (m, 3H), 3.00 (dd, *J* = 17.5, 7.7 Hz, 1H), 2.94 (dd, *J* = 13.7, 4.4 Hz, 1H), 2.75 (dd, *J* = 13.7, 10.4 Hz, 1H). ^{13}C NMR (126 MHz, DMSO) δ 172.13, 158.69, 158.40, 158.11, 157.82, 155.67, 149.49, 145.04, 138.24, 137.98, 136.61, 130.67, 130.40, 129.16, 128.23, 128.01, 127.59, 127.38, 127.15, 126.25, 80.09, 63.02, 56.33, 43.36, 41.12, 39.52, 37.47.

quinolin-3-ylmethyl ((S)-3-(benzo[b]thiophen-3-yl)-1-((((S)-3-bromo-4,5-dihydroisoxazol-5-yl)methyl)amino)-1-oxopropan-2-yl)carbamate, TFA salt (**7**):
(S)-3-(benzo[b]thiophen-3-yl)-2-((tert-butoxycarbonyl)amino)propanoic acid (0.386 g, 1.2 mmol) was dissolved in diethyl ether/methanol (2:1, 15 mL) and hydrochloric acid (4 M in dioxane) (0.600 mL, 2.400 mmol) was added. The mixture was cooled in an ice bath and TMS-Diazomethane (2 M in ether) (1.800 mL, 3.60 mmol) carefully added dropwise. The mixture was stirred for 30 minutes after which additional hydrochloric acid was added to destroy excess TMS-Diazomethane, which could easily be monitored by the disappearance of the characteristic yellow colour. The solvents were removed on the rotovap, yielding quantitative (S)-methyl 2-amino-3-(benzo[b]thiophen-3-yl)propanoate, HCl as a white solid that was used directly in the next step. The suitably protected intermediate was then elaborated to the final inhibitor using the method

outlined for compound **5**, furnishing quinolin-3-ylmethyl ((S)-3-(benzo[b]thiophen-3-yl)-1-((((S)-3-bromo-4,5-dihydroisoxazol-5-yl)methyl)amino)-1-oxopropan-2-yl)carbamate (30 mg, 0.053 mmol, 4.4 % yield over 4 steps) after preparative TLC. The product was further purified by reverse phase HPLC in water/acetonitrile with trifluoroacetic acid, and the final product obtained as its TFA salt. ^{1}H NMR (500 MHz, DMSO-d_6) δ 8.94 (d, *J* = 2.1 Hz, 1H), 8.47 (t, *J* = 6.0 Hz, 1H), 8.42 – 8.30 (m, 1H), 8.12 – 8.05 (m, 1H), 8.04 – 7.98 (m, 1H), 7.94 (d, *J* = 9.0 Hz, 2H), 7.84 (ddd, *J* = 8.4, 6.9, 1.5 Hz, 1H), 7.74 (d, *J* = 8.4 Hz, 1H), 7.70 (ddd, *J* = 8.1, 6.9, 1.2 Hz, 1H), 7.46 (s, 1H), 7.45 – 7.29 (m, 2H), 5.28 – 5.13 (m, 2H), 4.68 (ddt, *J* = 10.6, 7.2, 5.3 Hz, 1H), 4.40 (ddd, *J* = 10.0, 8.4, 4.8 Hz, 1H), 3.41 (dt, *J* = 14.1, 6.0 Hz, 1H), 3.31 (dd, *J* = 17.6, 10.7 Hz, 1H), 3.26 – 3.15 (m, 2H), 3.07 (dd, *J* = 14.4, 10.1 Hz, 1H), 2.97 (dd, *J* = 17.6, 7.2 Hz, 1H). ^{13}C NMR (126 MHz, DMSO-d_6) δ 172.02, 155.68, 149.66, 139.50, 138.58, 138.15, 136.24, 131.97, 130.50, 130.29, 128.20, 127.48, 127.44, 127.36, 124.24, 124.07, 123.99, 122.84, 121.86, 79.98, 63.15, 54.50, 43.49, 41.34, 39.52, 30.79.

quinolin-3-ylmethyl ((S)-1-((((S)-3-bromo-4,5-dihydroisoxazol-5-yl)methyl)amino)-3-(2-fluorophenyl)-1-oxopropan-2-yl)carbamate (8):
Compound **8** was prepared from (S)-2-((tert-butoxycarbonyl)amino)-3-(2-fluorophenyl)propanoic acid by the method used to prepare compound **7**. ^{1}H NMR (400 MHz, DMSO-d_6) δ 8.84 (d, *J* = 2.2 Hz, 1H), 8.32 (t, *J* = 5.9 Hz, 1H), 8.26 – 8.20 (m, 1H), 8.03 (d, *J* = 8.4 Hz, 1H), 7.99 – 7.93 (m, 1H), 7.77 (ddd, *J* = 8.5, 6.9, 1.5 Hz, 1H), 7.70 – 7.60 (m, 2H), 7.27 (td, *J* = 7.7, 1.8 Hz, 1H), 7.24 – 7.15 (m, 1H), 7.14 – 6.99 (m, 2H), 5.18 (s, 2H), 4.71 – 4.60 (m, 1H), 4.30 (td, *J* = 9.1, 5.3 Hz, 1H), 3.42 – 3.31 (m, 1H), 3.31 – 3.24 (m, 1H), 3.19 (dt, *J* = 14.0, 5.4 Hz, 1H), 3.02 (dd, *J* = 13.9, 5.6 Hz, 1H), 2.94 (dd, *J* = 17.5, 7.2 Hz, 1H), 2.82 (dd, *J* = 14.0, 9.7 Hz, 1H). HRMS (ESI-QTOF) m/z: calculated for $C_{24}H_{23}BrFN_4O_4^+$ [M+H]$^+$ 529.08812; found 529.08820

quinolin-3-ylmethyl ((S)-1-((((S)-3-bromo-4,5-dihydroisoxazol-5-yl)methyl)amino)-3-(4-fluorophenyl)-1-oxopropan-2-yl)carbamate (9):
Compound **9** was prepared from (S)-2-((tert-butoxycarbonyl)amino)-3-(4-fluorophenyl)propanoic acid by the method used to prepare compound **7**. ^{1}H NMR (400 MHz, Chloroform-*d*) δ 8.90 – 8.78 (m, 1H), 8.14 – 8.01 (m, 2H), 7.76 (d, *J* = 8.1 Hz, 1H), 7.70 (t, *J* = 7.7 Hz, 1H), 7.54 (t, *J* = 7.4 Hz, 1H), 7.09 (dd, *J* = 8.3, 5.1 Hz, 2H), 6.96 – 6.82 (m, 3H), 5.77 (d, *J* = 8.2 Hz, 1H), 5.28 – 5.13 (m, 2H), 4.72 (dd, *J* = 9.3, 4.8 Hz, 1H), 4.48 (q, *J* = 7.4 Hz, 1H), 3.45 (m, 2H), 3.16 (dd, *J* = 17.5, 10.6 Hz, 1H), 3.06 (dd, *J* = 13.9, 6.6 Hz, 1H), 2.96 (dd, *J* = 14.0, 7.6 Hz, 1H), 2.80 (dd, *J* = 17.5, 8.1 Hz, 1H). ^{13}C NMR (101 MHz, Chloroform-*d*) δ 171.91, 161.97 (d, *J* = 245.6 Hz), 155.82, 150.60, 147.72, 137.99, 135.81, 131.97 (d, *J* = 3.3 Hz), 130.81 (d, *J* = 8.0 Hz), 130.04, 129.15, 128.92, 127.99, 127.64, 127.18, 115.67 (d, *J* = 21.2 Hz), 80.32, 64.91, 56.47, 43.86, 41.68, 37.92. HRMS (ESI-QTOF) m/z: calculated for $C_{24}H_{23}BrFN_4O_4^+$ [M+H]$^+$ 529.08812; found 529.08816

quinolin-3-ylmethyl ((S)-1-((((S)-3-bromo-4,5-dihydroisoxazol-5-yl)methyl)amino)-3-(4-iodophenyl)-1-oxopropan-2-yl)carbamate (11):
Compound **11** was prepared from 4-iodophenylalanine analogous to the preparation of compound **7** (150 mg, 17.1% yield over 4 steps). ^{1}H NMR (500 MHz, DMSO-d_6) δ 9.02 (d, *J* = 1.9 Hz, 1H), 8.52 (d, *J* = 2.1 Hz, 1H), 8.43 (t, *J* = 6.0 Hz, 1H), 8.12 (d, *J* = 8.5 Hz, 1H), 8.08 (dd, *J* = 8.1, 1.3 Hz, 1H), 7.90 (ddd, *J* = 8.4, 6.0, 1.4 Hz, 1H), 7.80 – 7.66 (m, 2H), 7.63 – 7.55 (m, 2H), 7.13 – 7.02 (m, 2H), 5.32 – 5.14 (m, 2H), 4.79 – 4.61 (m, 1H), 4.31 – 4.16 (m, 1H), 3.44 – 3.28 (m, 2H), 3.23 (dt, *J* = 14.1, 5.2 Hz, 1H), 3.01 – 2.82 (m, 2H), 2.72 (dd, *J* = 13.6, 10.5 Hz, 1H). HRMS (ESI-QTOF) m/z: calculated for $C_{24}H_{23}BrIN_4O_4^+$ [M+H]$^+$ 636.99419; found 636.99407

tert-Butyl ((S)-1-((((S)-3-bromo-4,5-dihydroisoxazol-5-yl)methyl)amino)-3-(5-fluoro-1H-indol-3-yl)-1-oxopropan-2-yl)carbamate (14):
Commercial 5-fluoro-L-tryptophan hydrate (100 mg, 0.416 mmol) was dissolved in 5 mL THF-water (1:1) and 4-methylmorpholine (182 mg, 1.799 mmol) added, followed by Di-tert-butyl dicarbonate (1M in THF) (900 µL, 0.900 mmol) in a dropwise fashion. Reaction progress was monitored by TLC (20 % methanol in ethyl acetate) until all starting material had been consumed. Ether (10 mL) was added to the reaction mixture and the aqueous layer was withdrawn. The ether was extracted one additional time with water containing N-methylmorpholine. At this time, TLC of the ether layer showed no residual product.
The BOC protected amino acid was then directly used in the next step as the aqueous solution to which EDCI·HCl (96 mg, 0.501 mmol), 1H-benzo[d][1,2,3]triazol-1-ol (59 mg, 0.437 mmol) and (S)-(3-bromo-4,5-dihydroisoxazol-5-yl)methanamine (78 mg, 0.436 mmol) were added and the mixture stirred for 2 hours. The solution was then extracted with ethyl acetate three times and the combined organic layers were dried over sodium sulfate and evaporated, furnishing the crude product that was purified by preparative TLC, yielding the title compound (180 mg, 0.372 mmol, 90 % yield over two steps). ^{1}H NMR (500 MHz, DMSO-d_6) δ 10.91 (d, *J* = 2.6 Hz, 1H), 8.26 (t, *J* = 6.0 Hz, 1H), 7.41 (dd, *J* = 10.2, 2.6 Hz, 1H), 7.31 (dd, *J* = 8.8, 4.6 Hz, 1H), 7.23 (d, *J* = 2.4 Hz, 1H), 6.98 – 6.78 (m, 2H), 4.66 (ddt, *J* = 10.6, 7.5, 5.4 Hz, 1H), 4.14 (td, *J* = 9.0, 4.7 Hz, 1H), 3.39 (dt, *J* = 14.2, 6.0 Hz, 1H), 3.31 – 3.17 (m, 2H), 3.04 – 2.90 (m, 2H), 2.85 (dd, *J* = 14.5, 9.8 Hz, 1H), 1.29 (s, 9H).

quinoxalin-2-ylmethyl ((S)-1-((((S)-3-bromo-4,5-dihydroisoxazol-5-yl)methyl)amino)-3-(5-fluoro-1H-indol-3-yl)-1-oxopropan-2-yl)carbamate (12):

An aliquot of compound **14** (170 mg, 0.352 mmol) was taken up in a mixture of 2:1 DCM/TFA and stirred for 1 hour, after which aqueous sodium hydroxide was carefully added to reach a pH of 10-11. The mixture was extracted with DCM three times and the combined organic extracts were dried over sodium sulfate followed by evaporation of the volatiles to yield the deprotected product (S)-2-amino-N-(((S)-3-bromo-4,5-dihydroisoxazol-5-yl)methyl)-3-(5-fluoro-1H-indol-3-yl)propanamide (130 mg, 0.339 mmol, 96 % yield) as the free base that was used without further purification. An aliquot of (S)-2-amino-N-(((S)-3-bromo-4,5-dihydroisoxazol-5-yl)methyl)-3-(5-fluoro-1H-indol-3-yl)propanamide (35 mg, 0.091 mmol) and 4-nitrophenyl (quinoxalin-2-ylmethyl) carbonate (29.7 mg, 0.091 mmol) were dissolved in 3 mL DMF and N-Methylmorpholine (20 µl, 0.183 mmol) was added. The mixture was stirred for 18 hours and then diluted with water and extracted with ethyl acetate. The organic layer was washed with water and brine and then dried over sodium sulfate and finally evaporated. The crude product was purified by preparative TLC. ^{1}H NMR (400 MHz, DMSO-d_6) δ 10.94 (d, *J* = 2.3 Hz, 1H), 8.89 (s, 1H), 8.45 (t, *J* = 6.0 Hz, 1H), 8.15 – 8.07 (m, 1H), 8.06 – 7.99 (m, 1H), 7.91 – 7.82 (m, 2H), 7.76 (d, *J* = 8.2 Hz, 1H), 7.47 (dd, *J* = 10.3, 2.7 Hz, 1H), 7.32 (dd, *J* = 8.8, 4.6 Hz, 1H), 7.26 (d, *J* = 2.5 Hz, 1H), 6.89 (td, *J* = 9.2, 2.2 Hz, 1H), 5.37 – 5.17 (m, 2H), 4.66 (m, 1H), 4.25 (m, 1H), 3.46 – 3.15 (m, 3H), 3.08 – 2.83 (m, 3H).

quinolin-4-ylmethyl ((S)-1-((((S)-3-bromo-4,5-dihydroisoxazol-5-yl)methyl)amino)-3-(5-fluoro-1H-indol-3-yl)-1-oxopropan-2-yl)carbamate, TFA salt (13):
The title compound was synthesized by the method used to prepare compound **12** and purified by preparative TLC and reverse-phase HPLC in water/acetonitrile with trifluoroacetic acid, furnishing the final product as its TFA salt. ^{1}H NMR (500 MHz, DMSO-d_6) δ 10.96 (s, 1H), 8.94 (d, *J* = 4.8 Hz, 1H), 8.45 (s, 1H), 8.15 – 8.05 (m, 2H), 7.89 (t, *J* = 7.8 Hz, 1H), 7.80 (d, *J* = 8.6 Hz, 1H), 7.75 – 7.68 (m, 1H), 7.52 – 7.45 (m, 1H), 7.39 (d, *J* = 4.8 Hz, 1H), 7.34 (dd, *J* = 8.8, 4.6 Hz, 1H), 7.28 (d, *J* = 2.4 Hz, 1H), 6.90 (td, *J* = 9.2, 2.6 Hz, 1H), 5.57 (s, 2H), 4.76 – 4.59 (m, 1H), 4.37 – 4.24 (m, 1H), 3.42 (dt, *J* = 14.1, 6.0 Hz, 1H), 3.30 (dd, *J* = 17.6, 10.8 Hz, 1H), 3.23 (dt, *J* = 14.0, 5.3 Hz, 1H), 3.03 (dd, *J* = 14.5, 4.7 Hz, 1H), 3.00 – 2.87 (m, 2H). HRMS (ESI-QTOF) m/z: calculated for $C_{26}H_{24}BrFN_5O_4^+$ $[M+H]^+$ 568.09902; found 568.09873

4-nitrophenyl (quinolin-3-ylmethyl) carbonate (S1):
The title compound was prepared as described in the literature. (Watts et al., 2006)

4-nitrophenyl (quinolin-4-ylmethyl) carbonate (S2):
Similar to the published example for the 3-quinolyl isomer (Watts et al., 2006), commercial quinoline-4-carbaldehyde (3 g, 19.09 mmol) was dissolved in absolute ethanol (40 mL) and cooled in a dry-ice acetone bath. Then, Lithium borohydride (2 M in THF) was added dropwise and the reaction monitored by TLC until full conversion of the starting material could be observed. At that time, the cooling bath was removed and 1 M aqueous HCl carefully added. Once the reaction had reached room temperature and additional HCl would not yield more gas evolution, aqueous sodium bicarbonate was added to reach a neutral pH. The reaction mixture was concentrated under reduced pressure before the aqueous mixture was extracted with ethyl acetate three times and the combined organic layers were dried over sodium sulfate and evaporated, yielding the alcohol as an orange oil that solidified on standing. The alcohol was taken up in anhydrous DCM (30 mL), N-Methylmorpholine (4.20 ml, 38.2 mmol) was added and the solution cooled in an ice-bath. Separately, 4-nitrophenyl chloroformate (5.77 g, 28.6 mmol) was dissolved in DCM (15 mL) and added dropwise to the cooled solution of the alcohol. After addition had been completed, the mixture was allowed to warm to room temperature and stirred overnight. Then, aqueous sodium bicarbonate and ethyl acetate were added to the reaction mixture. The organic layer was washed with sodium bicarbonate solution (3x) and brine. The organic phase was dried over sodium sulfate and evaporated, yielding a red crude product that solidified on standing. The crude product was purified by silica gel chromatography and eluted using a gradient of pentane in ethyl acetate (20% --> 50%), affording the title compound (3.1 g, 9.56 mmol, 50.1 % yield) as an off-white solid. ^{1}H NMR (400 MHz, Chloroform-*d*) δ 8.97 (d, *J* = 4.4 Hz, 1H), 8.35 – 8.26 (m, 2H), 8.24 – 8.13 (m, 1H), 8.01 (dt, *J* = 8.4, 0.9 Hz, 1H), 7.80 (ddd, *J* = 8.4, 6.8, 1.4 Hz, 1H), 7.67 (ddd, *J* = 8.3, 6.9, 1.3 Hz, 1H), 7.55 (d, *J* = 4.4 Hz, 1H), 7.41 (d, *J* = 9.2 Hz, 2H), 5.80 (d, *J* = 0.8 Hz, 2H).

4-nitrophenyl (quinoxalin-2-ylmethyl) carbonate (S3):
Inspired by the work of Robinson and Taylor (Robinson & Taylor, 2005), benzene-1,2-diamine (1.5 g, 13.87 mmol) and dihydroxyacetone (1.249 g, 13.87 mmol) were dissolved in DMSO (15 mL) and the solution heated to 80 °C. Then, 10 mol% triethylamine (0.193 ml, 1.387 mmol) was added, followed by 2 mol% Palladium(II) acetate (0.062 g, 0.277 mmol) . The mixture was stirred and heated while open to air. After 3 hours, the mixture was diluted with water and extracted with ethyl acetate. The organic layer was washed four times with a buffer of phosphoric acid and monobasic sodium phosphate (pH = 2) to remove amines followed by aqueous sodium bicarbonate. Evaporation of the organic solvent and purification of the crude material by silica gel chromatography afforded the desired quinoxalin-2-ylmethanol in a moderate yield.The alcohol was then coupled to 4-nitrophenyl chloroformate using the procedure outlined for compound **S2**, furnishing the title compound in modest overall yield (20 % over two steps). ^{1}H NMR (400 MHz, Chloroform-*d*) δ 9.02 (s, 1H), 8.36 – 8.25 (m, 2H), 8.22 – 8.09 (m, 2H), 7.87 – 7.76 (m, 2H), 7.51 – 7.39 (m, 2H), 5.64 (s, 2H).

(S)-(3-bromo-4,5-dihydroisoxazol-5-yl)methanamine (S-DHI)

The (S)-DHI building block was prepared using our modification (DiRaimondo et al., 2014) to the [3+2] dipolar cycloaddition reaction (Rohlloff, Robinson III, & Gardner, 1992) and fractional crystallization (Castelhano, Billedeau, Pliura, Bonaventura, & Krantz, 1988) reported in the literature.

Bibliography

Castelhano, A. L., Billedeau, R., Pliura, D. H., Bonaventura, B. J., & Krantz, A. (1988). Synthesis, chemistry, and absolute configuration of novel transglutaminase inhibitors containing a 3-halo-4,5-dihydroisoxazole. *Bioorganic Chemistry*, *16*(3), 335–340. doi:10.1016/0045-2068(88)90019-3

Dafik, L., & Khosla, C. (2011). Dihydroisoxazole Analogs for Labeling and Visualization of Catalytically Active Transglutaminase 2. *Chem. Biol.*, *18*(1), 58–66. doi:10.1016/j.chembiol.2010.11.004

DiRaimondo, T. R., Klöck, C., Warburton, R., Herrera, Z., Penumatsa, K., Toksoz, D., … Fanburg, B. (2014). Elevated transglutaminase 2 activity is associated with hypoxia-induced experimental pulmonary hypertension in mice. *ACS Chem. Biol.*, *9*(1), 266–75. doi:10.1021/cb4006408

Robinson, R. S., & Taylor, R. J. (2005). Quinoxaline Synthesis from α-Hydroxy Ketones via a Tandem Oxidation Process Using Catalysed Aerobic Oxidation. *Synlett*, (6), 1003–1005. doi:10.1055/s-2005-864830

Rohlloff, J. C., Robinson III, J., & Gardner, J. O. (1992). Bromonitrile oxide [3+2] cycloadditions in water. *Tetrahedron Lett.*, *33*(22), 3113–3116. doi:10.1016/S0040-4039(00)79827-3

Watts, R. E., Siegel, M., & Khosla, C. (2006). Structure-Activity Relationship Analysis of the Selective Inhibition of Transglutaminase 2 by Dihydroisoxazoles. *J. Med. Chem.*, *49*(25), 7493–7501. doi:10.1021/jm060839a

NMR spectra of compound **5**:

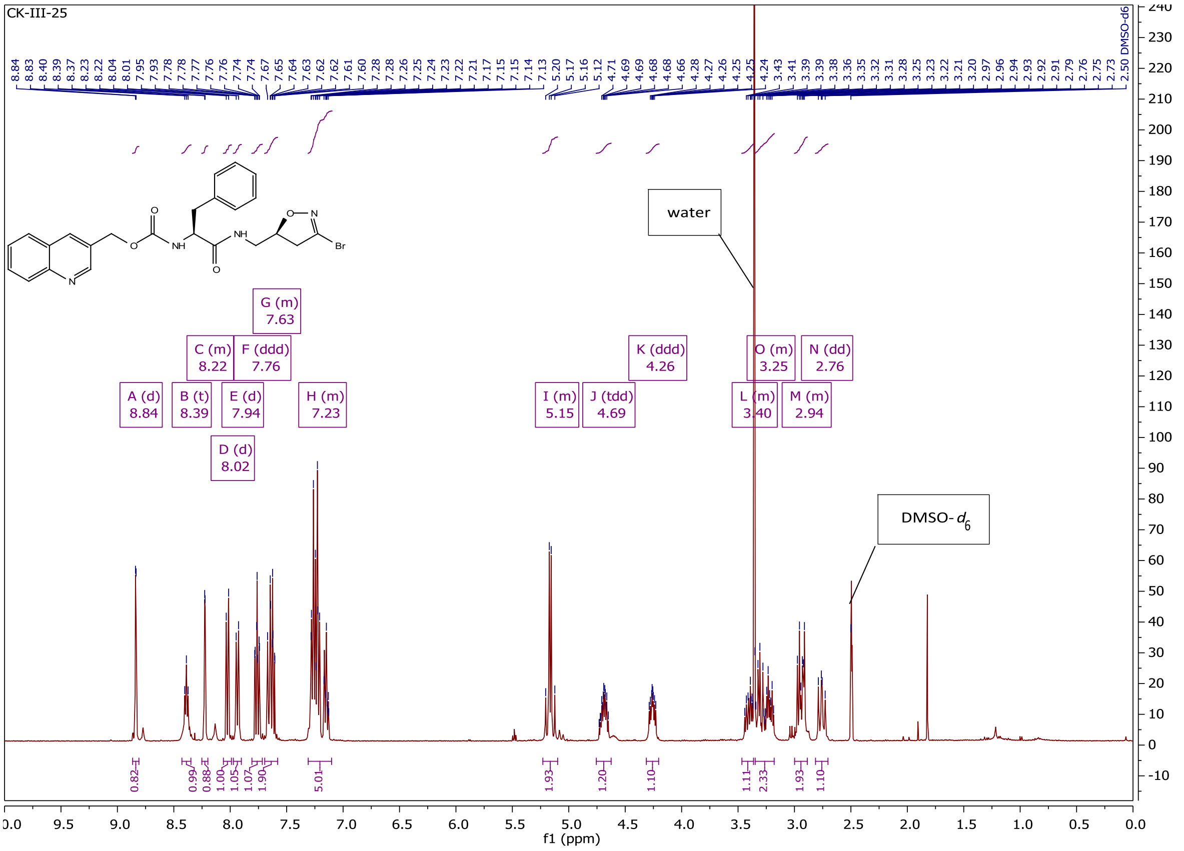

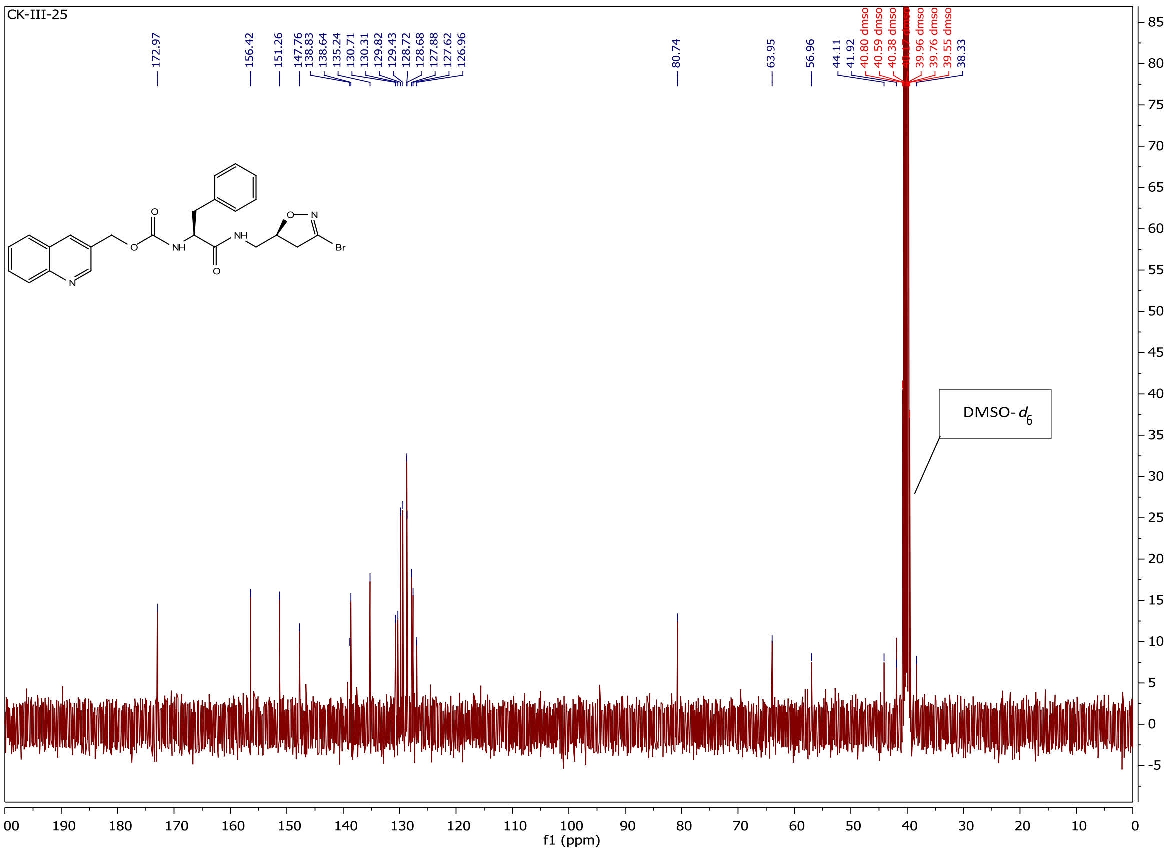

NMR spectra of compound **6**:

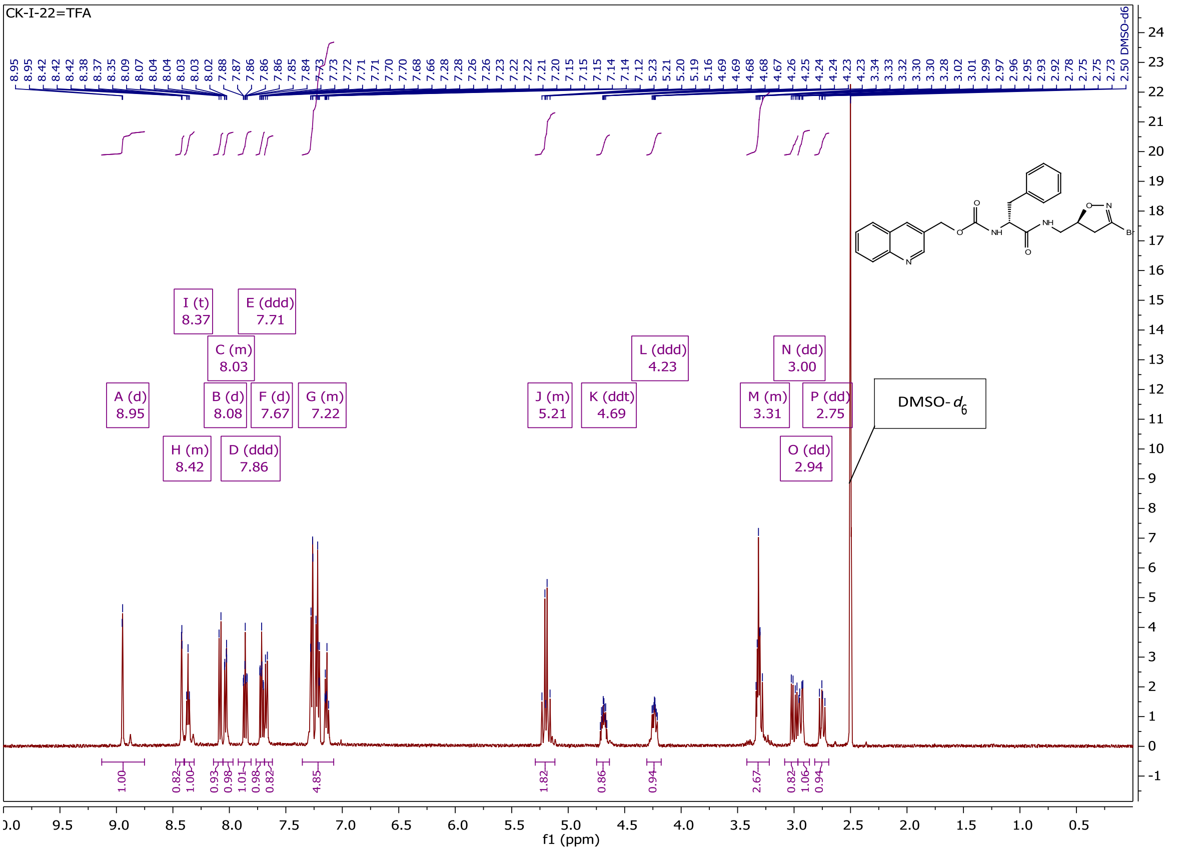

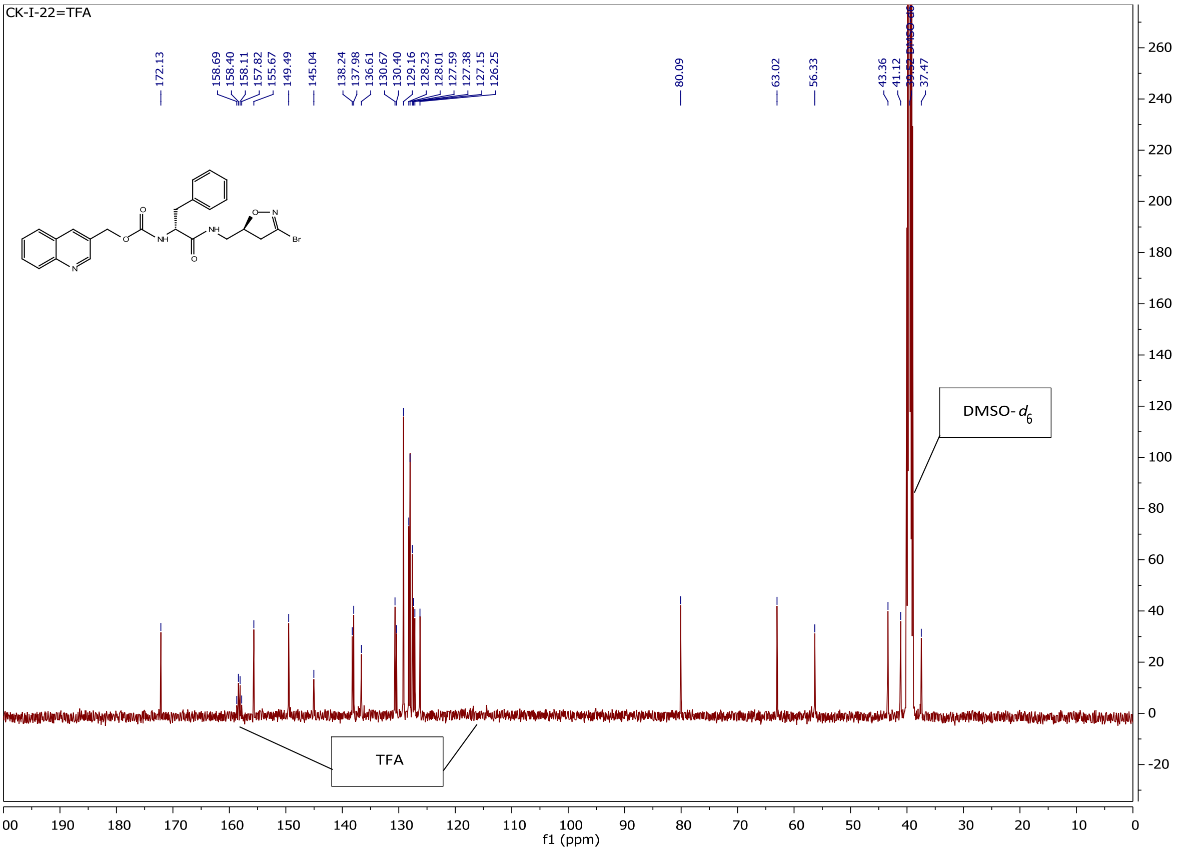

NMR spectra of compound **7**:

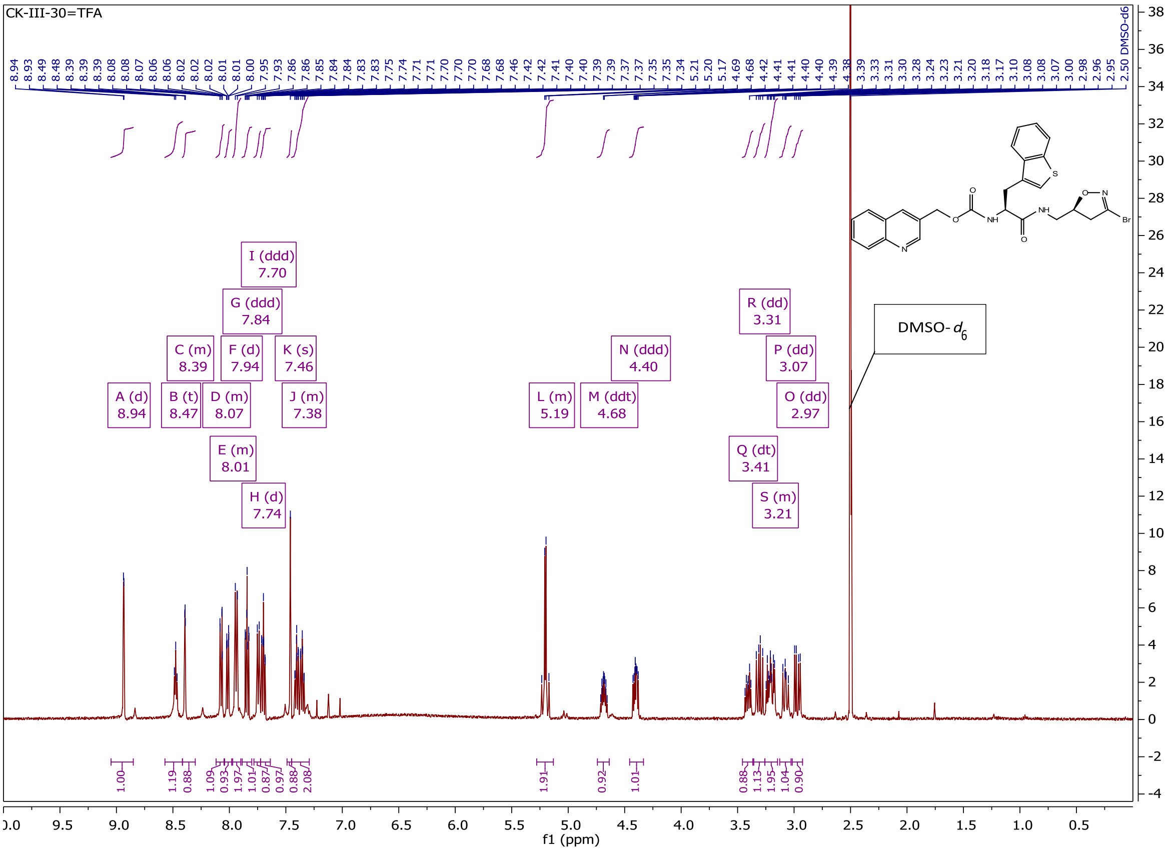

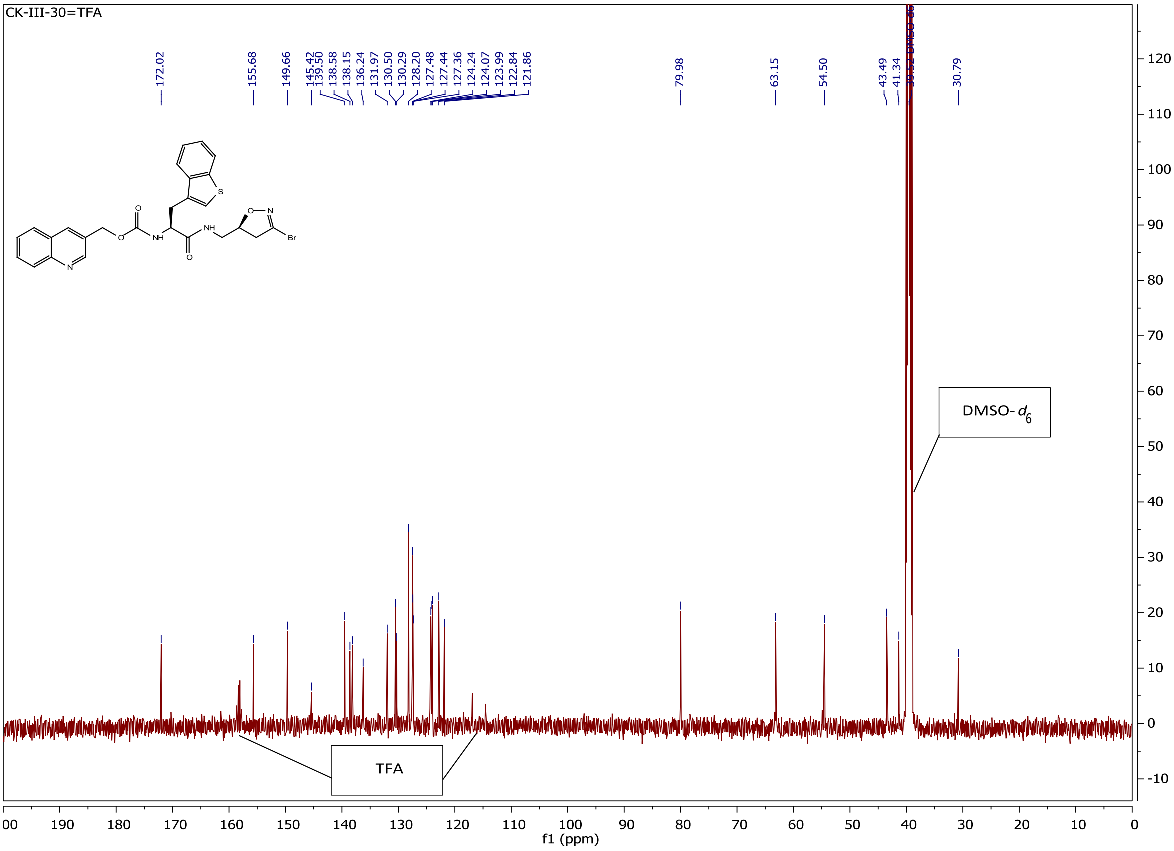

NMR spectra of compound **8**:

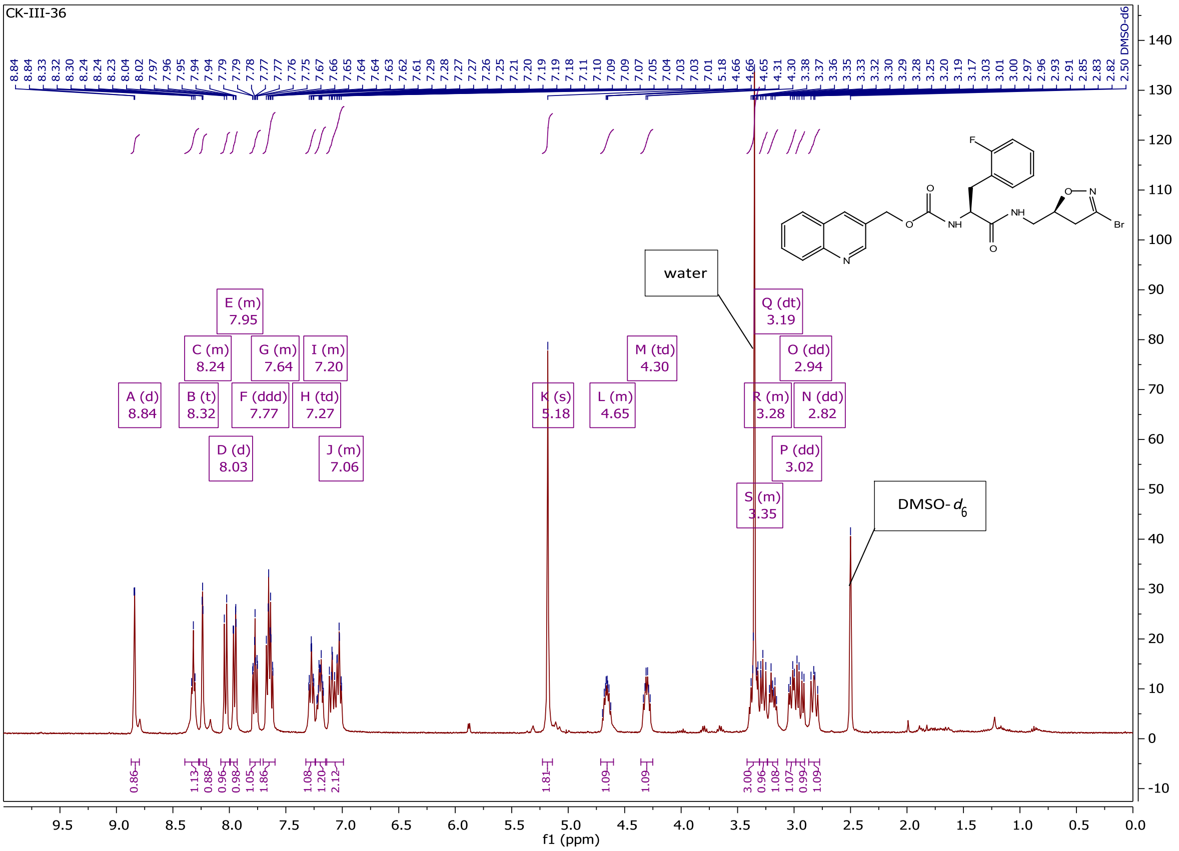

NMR spectra of compound **9**:

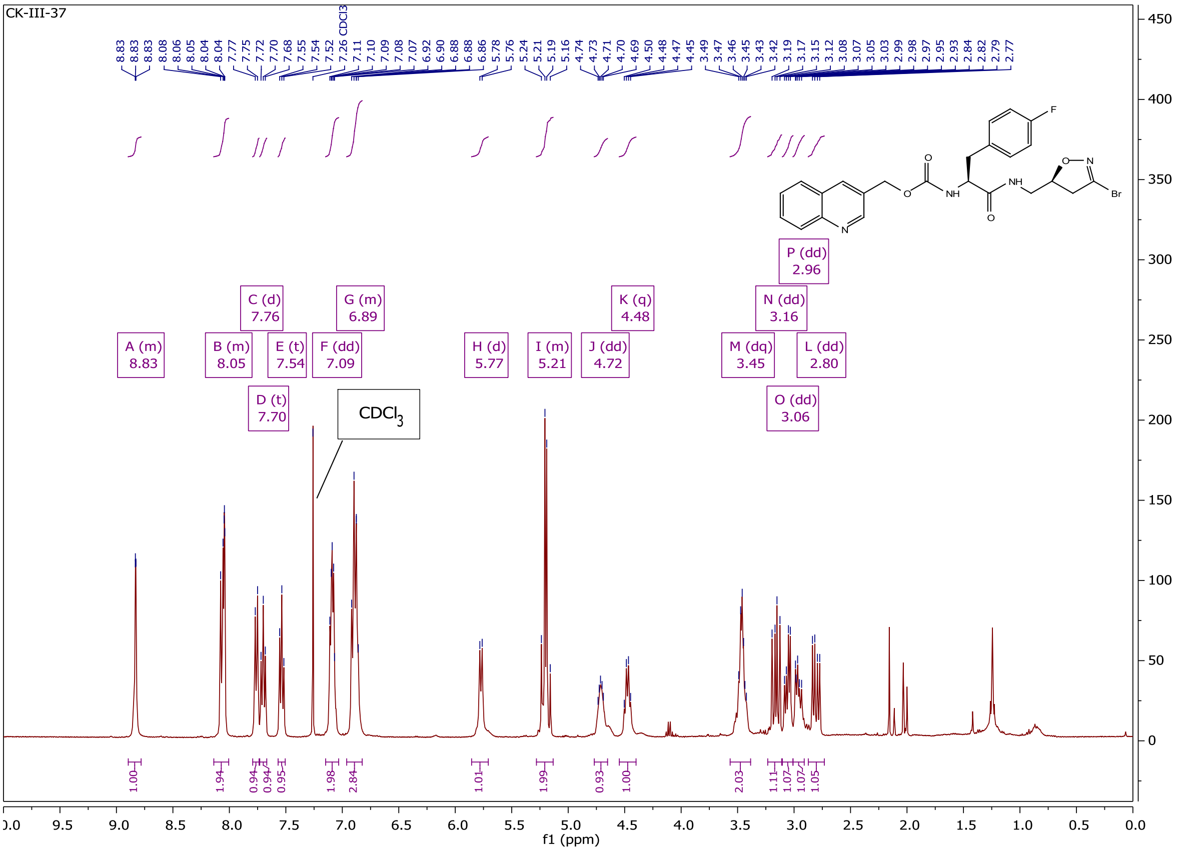

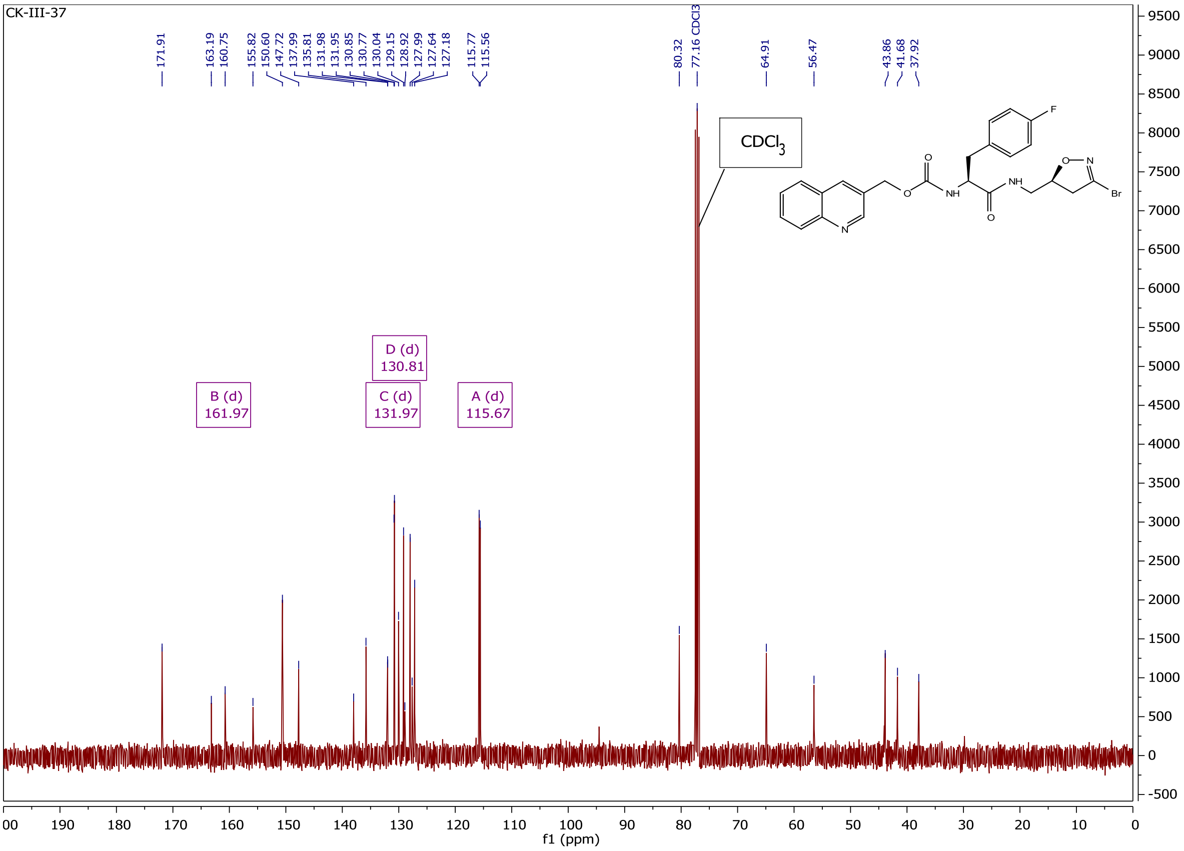

NMR spectra of compound **11**:

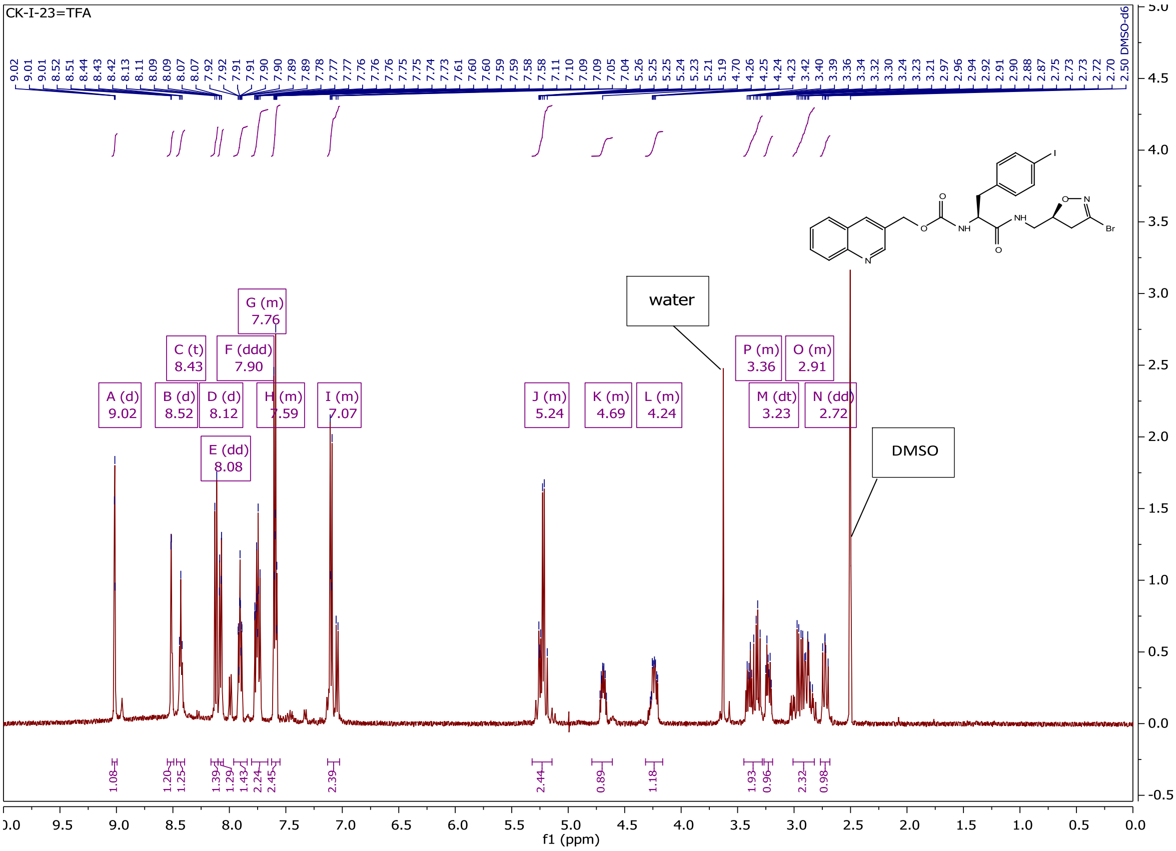

NMR spectra of compound **12**:

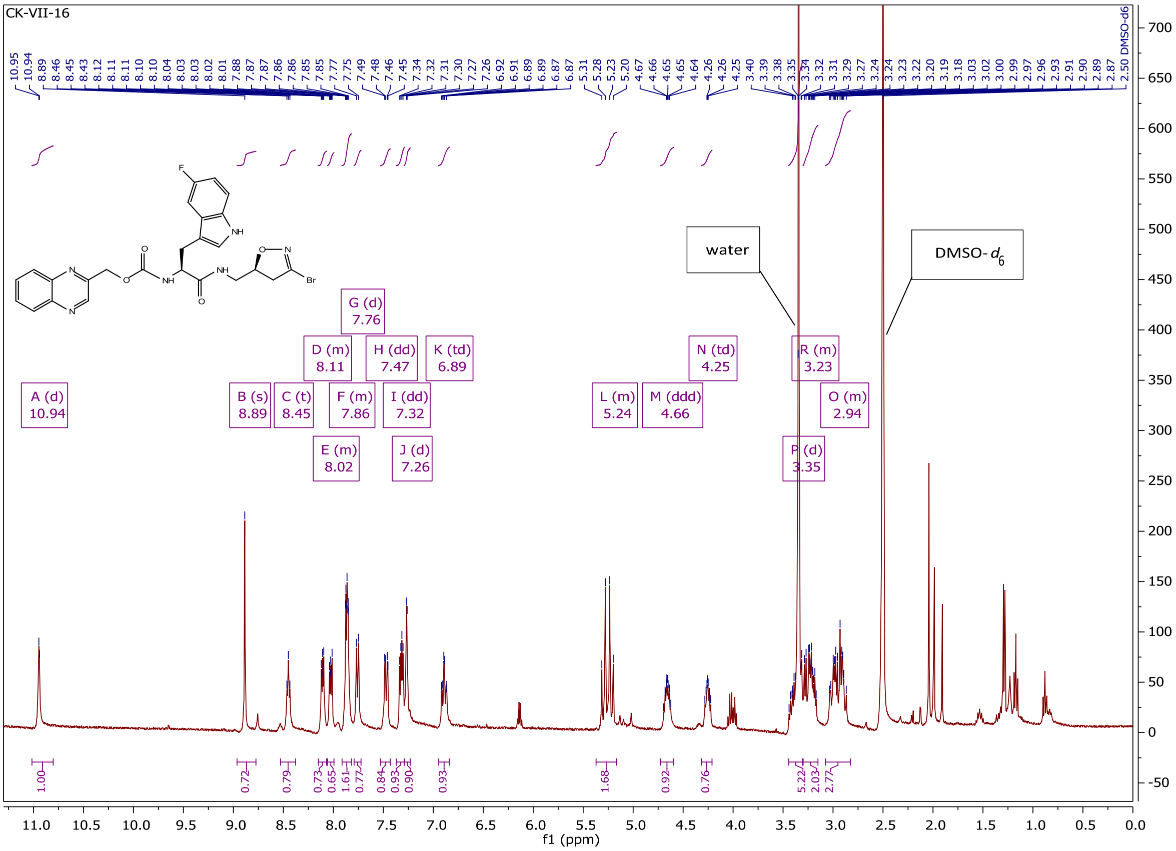

NMR spectra of compound **13**:

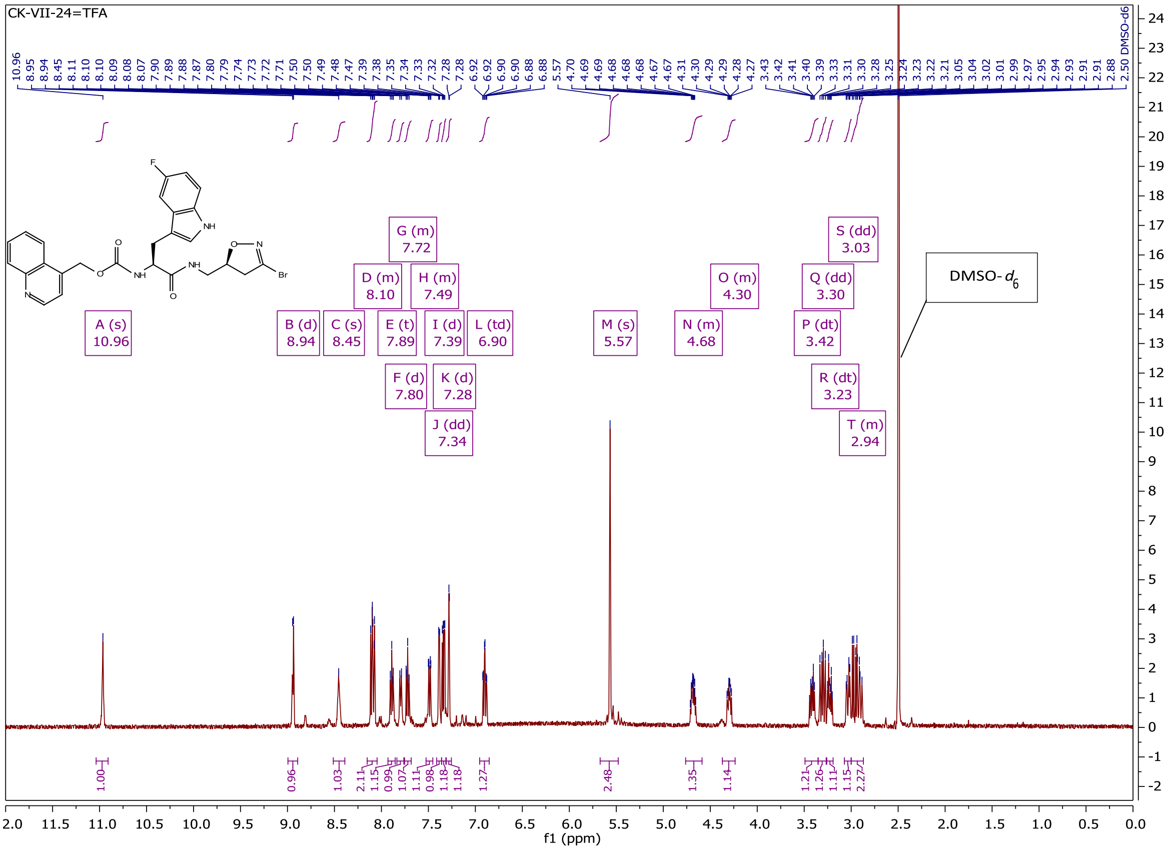

NMR spectra of compound **14**:

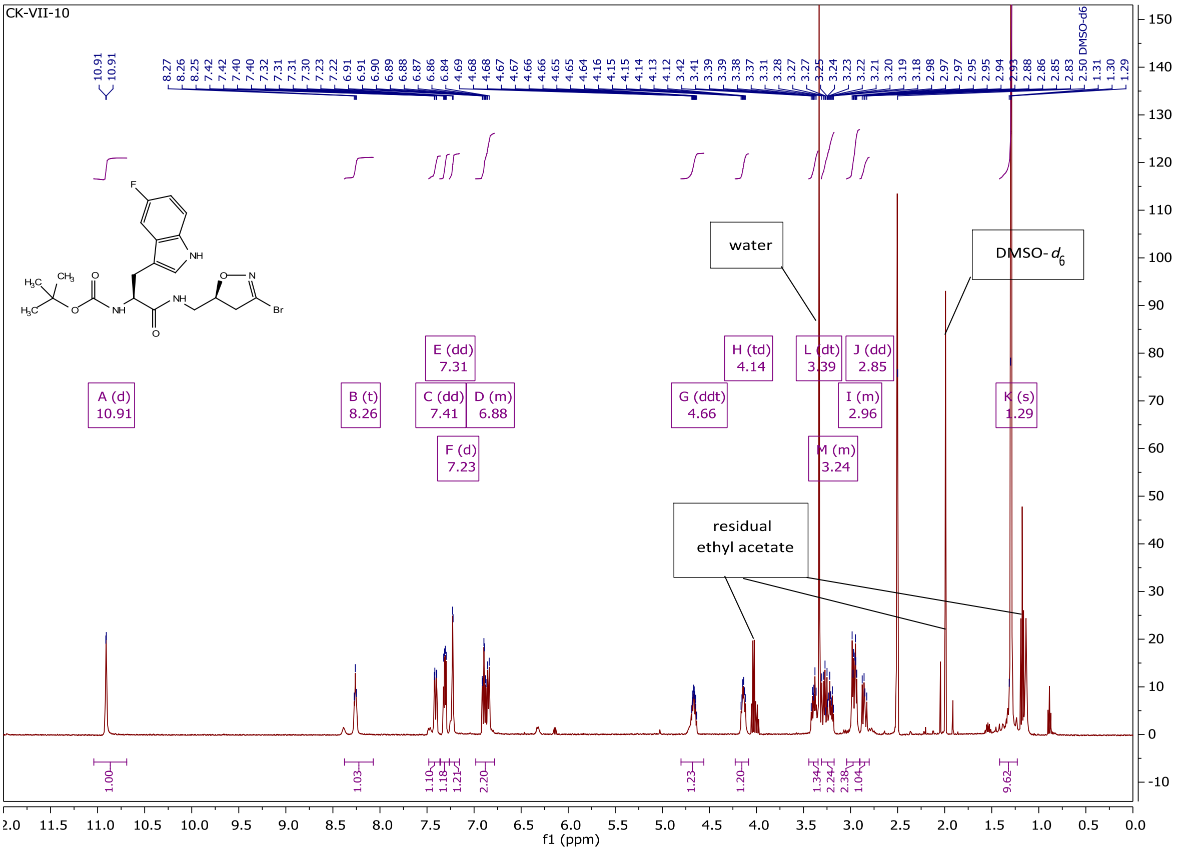

Supplement: Additional file 1 — Chemical Synthesis. [file 1475-2875-13-210-S1.pdf]
