# Supplementary material for: Dihydroisoxazole inhibitors of Anopheles gambiae seminal transglutaminase AgTG3
Source: Malar J. 2014 Jun 2;13:210. doi: 10.1186/1475-2875-13-210 (PMC4113009; doi:10.1186/1475-2875-13-210)
Supplement: Additional file 2: Table S1 — TG inhibitor library structures. [file 1475-2875-13-210-S2.pdf]

Supplementary Table 1. TG Inhibitor Library Structures

The library consisted of 92 transglutaminase inhibitors, comprising the following chemotypes:

- 83 dihydroisoxazole (DHI) inhibitors (labeled DXX)
- 8 acylidene oxoindoles (labeled IXX)
- 1 thienopyrimidinones (labeled MXX)

The DHI inhibitors were based on the 3-halo-4,5-dihydroisoxazole motive that covalently and irreversibly inactivates the active site of transglutaminases.[1-7] The acylidene oxoindoles appear as reversible, slow tight binding inhibitors of TG2; their (allosteric) binding site is unknown.[8] The thienopyrimidinones are also allosteric inhibitors with an unknown binding site on TG2.[9, 10] All inhibitors were dissolved to 10 mM in DMSO to a final volume of 60–100 μL and stored at -20 °C until use. The DMSO stock solutions were diluted to 100 μM in aqueous buffer for experiments. The table is a numbered list of compounds assayed inhibition for AgTG3, with internal compound code, SMILES string, chemical structure and reference. Footnotes refer to unpublished work of the following Khosla laboratory researchers: * Kihang Choi, † Eun Cho, ‡ Cornelius Klöck, § Edward Watts.

Supplementary References

1. Castelhano AL, Billedeau R, Pliura DH, Bonaventura BJ, Krantz A: **Synthesis, chemistry, and absolute configuration of novel transglutaminase inhibitors containing a 3-halo-4,5-dihydroisoxazole.** *Bioorganic Chemistry* 1988, **16:**335-340.
2. Hausch F, Halttunen T, Maki M, Khosla C: **Design, synthesis, and evaluation of gluten peptide analogs as selective inhibitors of human tissue transglutaminase.** *Chem Biol* 2003, **10:**225-231.
3. Choi K, Siegel M, Piper JL, Yuan L, Cho E, Strnad P, Omary B, Rich KM, Khosla C: **Chemistry and biology of dihydroisoxazole derivatives: selective inhibitors of human transglutaminase 2.** *Chem Biol* 2005, **12:**469-475.
4. Watts RE, Siegel M, Khosla C: **Structure-activity relationship analysis of the selective inhibition of transglutaminase 2 by dihydroisoxazoles.** *J Med Chem* 2006, **49:**7493-7501.
5. Dafik L, Albertelli M, Stamnaes J, Sollid LM, Khosla C: **Activation and inhibition of transglutaminase 2 in mice.** *PLoS One* 2012, **7:**e30642.
6. Diraimondo TR, Jin X, Klöck C, Khosla C: **Modulation of tissue transglutaminase activation in disease.** U.S. Patent Appl. WO2012177640 A3, Mar 14, 2013.
7. Diraimondo TR, Klöck C, Warburton R, Herrera Z, Penumatsa K, Toksoz D, Hill N, Khosla C, Fanburg B: **Elevated transglutaminase 2 activity is associated with hypoxia-induced experimental pulmonary hypertension in mice.** *ACS Chem Biol* 2014, **9:**266-275.
8. Klöck C, Jin X, Choi K, Khosla C, Madrid PB, Spencer A, Raimundo BC, Boardman P, Lanza G, Griffin JH: **Acylideneoxoindoles: a new class of reversible inhibitors of human transglutaminase 2.** *Bioorg Med Chem Lett* 2011, **21:**2692-2696.
9. Duval E, Case A, Stein RL, Cuny GD: **Structure-activity relationship study of novel tissue transglutaminase inhibitors.** *Bioorg Med Chem Lett* 2005, **15:**1885-1889.
10. Case A, Stein RL: **Kinetic analysis of the interaction of tissue transglutaminase with a nonpeptidic slow-binding inhibitor.** *Biochemistry* 2007, **46:**1106-1115.

#	ID	SMILES	Structure	Ref.
1	**D11**	ClC1=NO[C@@H](C1)[C@@H](C(O)=O)NC(OCC2C(C=CC=C3)=C3C4=C2C=CC=C4)=O	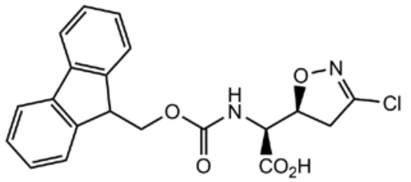	[2]
2	**D12**	BrC1=NOC(C1)CNC([C@H](CC2=CC=CC=C2)NC(OCC3=CC=CC=C3)=O)=O	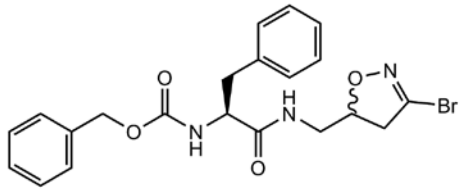	[3]
3	**D13**	BrC1=NOC(C1)CNC(CC[C@@H](C(OC)=O)NC(OCC2=CC=CC=C2)=O)=O	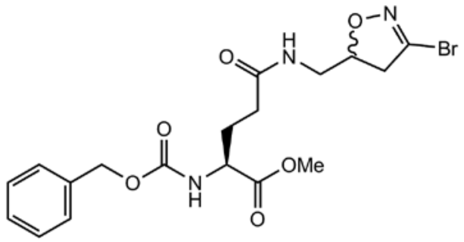	*
4	**D14**	BrC1=NOC(C1)CNC(C[C@@H](C(OC)=O)NC(OCC2=CC=CC=C2)=O)=O	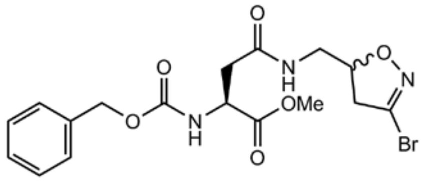	*
5	**D15**		Ac-PQPE(DHI)LPF-NH_2	[2]
6	**D16**	BrC1=NOC(CNC([C@H](CC(OC(C)(C)C)=O)NC(OCC2=CC=CC=C2)=O)=O)C1	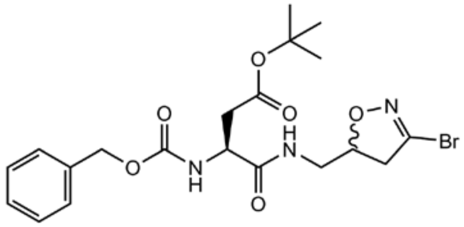	*
7	**D17**		Z-FD(DHI)LP-NH-CH_2-CH_2-Ph	*
8	**D21**	BrC1=NOC(CNC([C@H](C)NC(OCC2=CC=CC=C2)=O)=O)C1	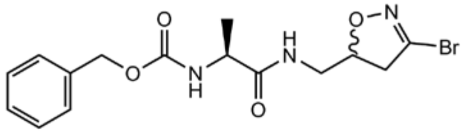	[3]
9	**D22**	BrC1=NOC(CNC([C@H](CC2=CC=CC=C2)NC(C)=O)=O)C1	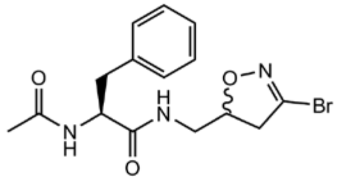	[3]

10	**D23**	BrC1=NOC(CNC([C@@H](CC2=CC=CC=C2)NC(OCC3=CC=CC=C3)=O)=O)C1	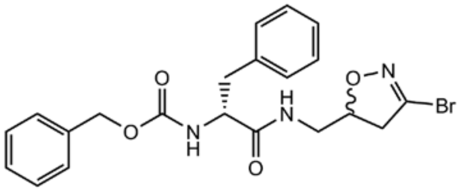	[3]
11	**D24**	BrC1=NOC(CNC([C@H](CC(O)=O)NC(OCC2=CC=CC=C2)=O)=O)C1	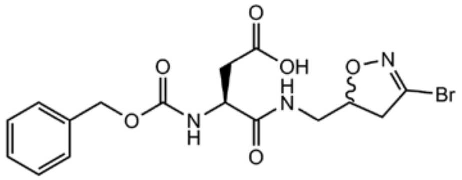	*
12	**D25**	BrC1=NOC(CNC([C@H](CC2=CC=CC=C2)NC(OCC3=CC=CC=C3)=O)=O)C1	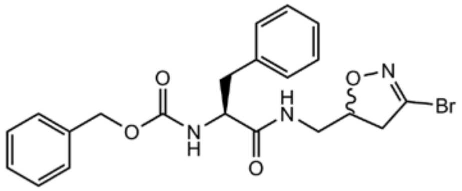	[3]
13	**D26**	BrC1=NO[C@@H](CNC([C@H](CC2=CC=C(O)C=C2)NC(OCC3=CC=CC=C3)=O)=O)C1	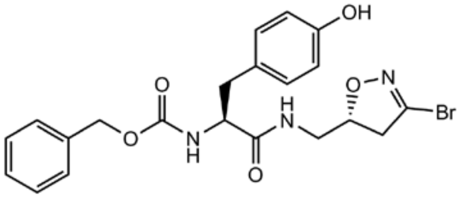	[4]
14	**D27**	BrC1=NO[C@H](CNC([C@H](CC2=CC=C(O)C=C2)NC(OCC3=CC=CC=C3)=O)=O)C1	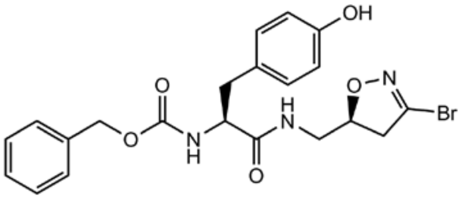	[4]
15	**D28**	BrC1=NOC(CNC(C(CC2=CNC3C2C=C(O)C=C3)NC(OCC4=CC=CC=C4)=O)=O)C1	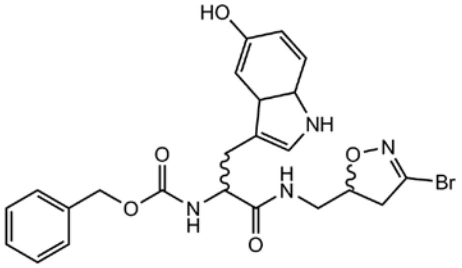	[3]
16	**D31**	BrC1=NOC(CNC([C@H](CC2=CC=C(F)C=C2)NC(OCC3=CC=CC=C3)=O)=O)C1	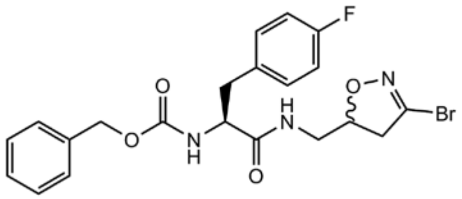	[3]

17	**D32**	BrC1=NOC(CNC([C@H](CC2=CC=CC(F)=C2)NC(OCC3=CC=CC=C3)=O)=O)C1	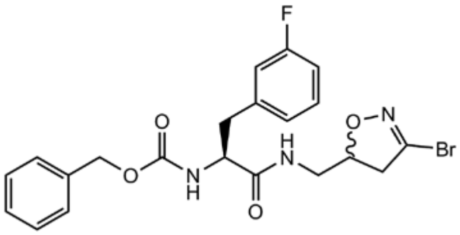	[3]
18	**D33**	BrC1=NOC(CNC([C@H](CC2=CC=C(O)C=C2)NC(OCCN(C(C3=C4C=CC=C3)=O)S4(=O)=O)=O)=O)C1	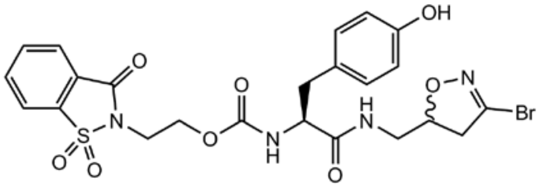	*
19	**D34**	BrC1=NOC(CNC([C@H](CC2=CC=C(O)C=C2)NC(OCC3=CC4=C(C=CC=C4)S3)=O)=O)C1	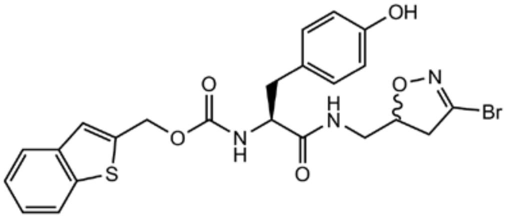	*
20	**D41**	BrC1=NOC(CNC([C@H](CC2=CC=C(O)C=C2)NC(OCC3=CC=CN=C3)=O)=O)C1	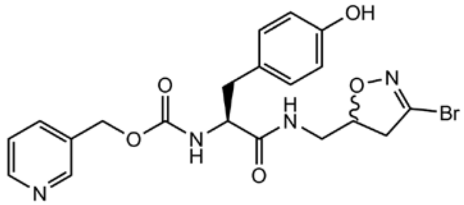	[3]
21	**D42**	BrC1=NOC(CNC([C@H](CC2=CC=C(O)C=C2)NC(OCC3=CC(C=CC=C4)=C4C=C3)=O)=O)C1	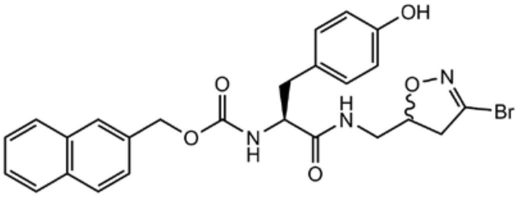	[3]
22	**D43**	BrC1=NOC(CNC([C@H](CC2=CC=C(O)C=C2)NC(OCCC3=CC=CC=C3)=O)=O)C1	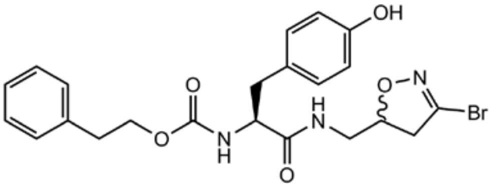	[3]
23	**D45**	BrC1=NOC(CNC([C@H](CC2=CC=C(O)C=C2)NC(OCCS(C3=CC=CC=C3)(=O)=O)=O)=O)C1	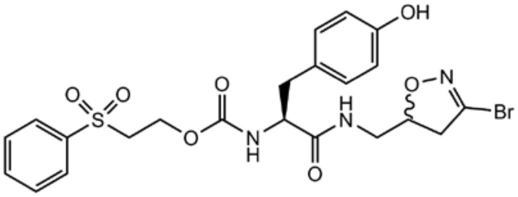	†

24	**D51**	BrC1=NOC(CNC(C(CC2=CNC3C2C=C(F)C=C3)NC(OCC4=CC=CC=C4)=O)=O)C1	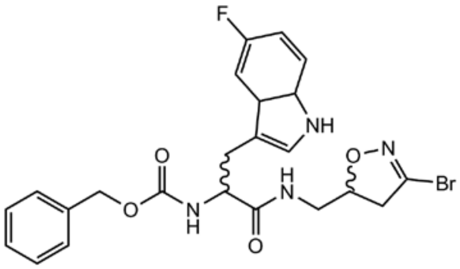	[4]
25	**D52**	BrC1=NOC(CNC(C(CC2=CNC3C2C(F)=CC=C3)NC(OCC4=CC=CC=C4)=O)=O)C1	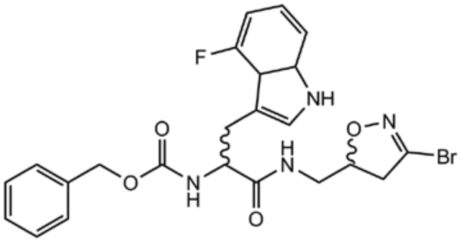	[4]
26	**D53**	BrC1=NOC(CNC(C(CC2=CNC3C2C=CC(F)=C3)NC(OCC4=CC=CC=C4)=O)=O)C1	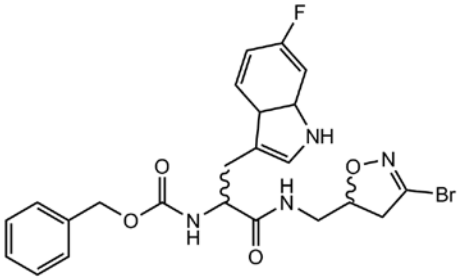	[4]
27	**D54**	BrC1=NOC(CNC(C(CC2=CNC3C2C=CC=N3)NC(OCC4=CC=CC=C4)=O)=O)C1	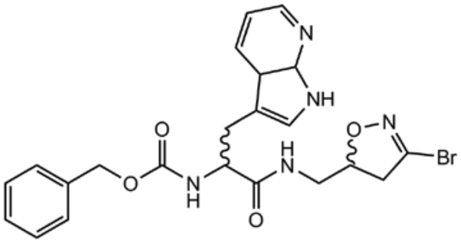	[4]
28	**D55**	BrC1=NO[C@H](CNC([C@H](CCC2)N2C(OCC3=CC(C=CC=C4)=C4N=C3)=O)=O)C1	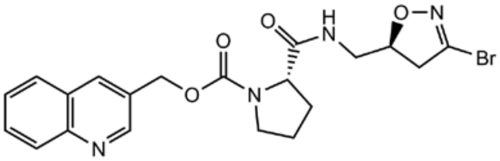	[4]
29	**D56**	BrC1=NO[C@H](CNC([C@H](CC2=CNC3C2C=C(F)C=C3)NC(OCC4=CC(C=CC=C5)=C5N=C4)=O)=O)C1	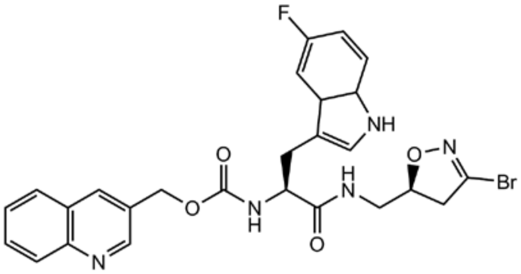	[4]

30	**D57**	BrC1=NOC(CNC(C(CC2=CNC3C2C=C(F)C=C3)NC(OCC4CNC5=C(C=CC=C5)O4)=O)=O)C1	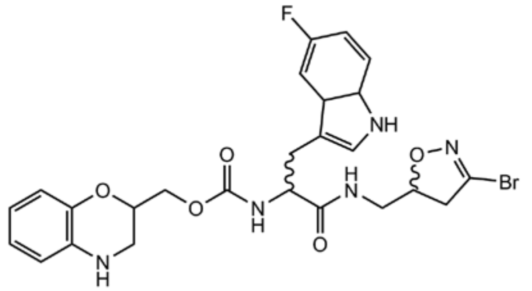	§
31	**D58**	BrC1=NOC(CNC([C@H](CC2=CC=C(O)C=C2)NC(OCC3CNC4=C(C=CC=C4)O3)=O)=O)C1	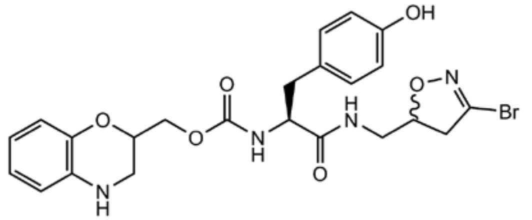	§
32	**D61**	BrC1=NO[C@H](CNC([C@H](CC2=CC=C(O)C=C2)NC(OCC3=CC(C=CC=C4)=C4N=C3)=O)=O)C1	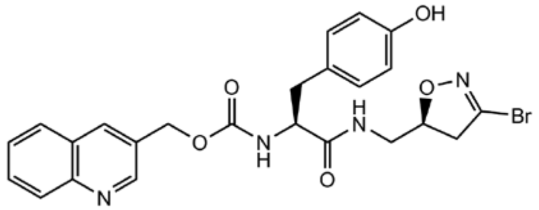	[4]
33	**D62**	BrC1=NO[C@@H](CNC([C@H](CC2=CC=C(O)C=C2)NC(OCC3=CC(C=CC=C4)=C4N=C3)=O)=O)C1	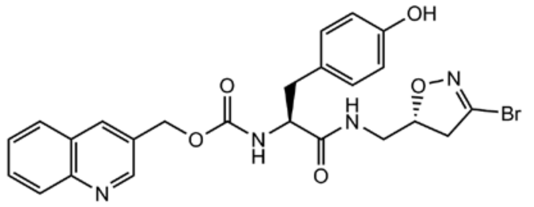	[4]
34	**D63**	BrC1=NO[C@@H](CNC([C@H]2N(CCC2)C(OCC3=CC(C=CC=C4)=C4N=C3)=O)=O)C1	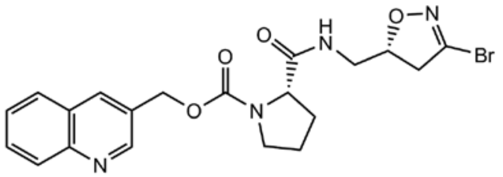	[4]
35	**D64**	BrC1=NO[C@H](CNC([C@H]2N(CCC2)C(OCC3=CC(C=CC=C4)=C4N=C3Cl)=O)=O)C1	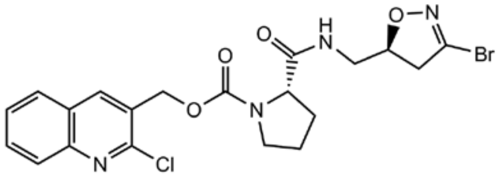	§
36	**D65**	BrC1=NO[C@@H](CNC([C@H]2N(CCC2)C(OCC3=CC(C=CC=C4)=C4N=C3Cl)=O)=O)C1	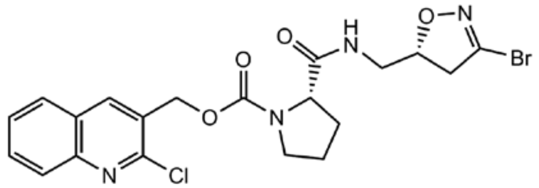	§

37	**D66**	BrC1=NO[C@@H](CNC([C@H](CC2=CNC3C2C=C(F)C=C3)NC(OCC4=CC(C=CC=C5)=C5N=C4)=O)=O)C1	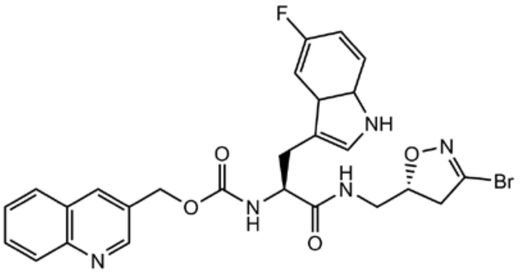	[4]
38	**D71**	BrC1=NO[C@@H](CNC([C@@H](NC(OCC2C3=C(C4=C2C=CC=C4)C=CC=C3)=O)CC5=CC=C(O)C=C5)=O)C1	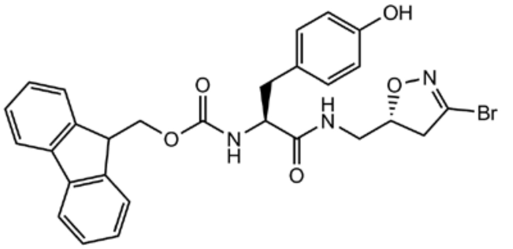	§
39	**D72**	O=C(N[C@H](C(N1[C@@H](CCC1)C(NC[C@H]2ON=C(Br)C2)=O)=O)CCC(N)=O)OCC3=CC=CC=C3	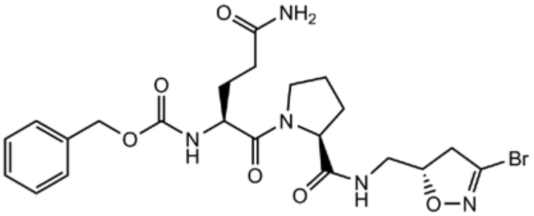	§
40	**D73**	BrC1=NO[C@H](CNC([C@@H](NC(OCC2C3=C(C4=C2C=CC=C4)C=CC=C3)=O)CC5=CC=C(O)C=C5)=O)C1	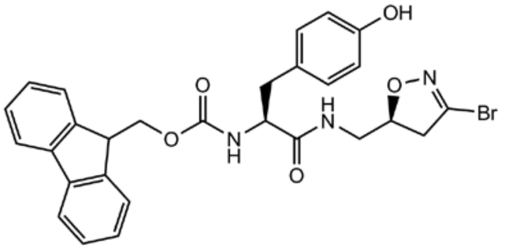	§
41	**D81**	BrC1=NO[C@H](CNC([C@@H](CC2=CC=CC=C2)NC(OCC3=CC(C=CC=C4)=C4N=C3)=O)=O)C1	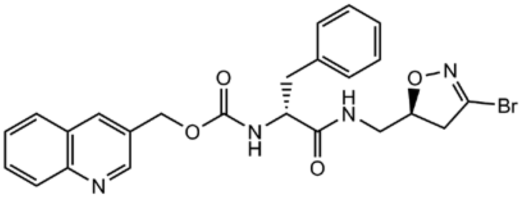	This work
42	**D82**	BrC1=NO[C@H](CNC([C@H](CC2=CC=C(I)C=C2)NC(OCC3=CC(C=CC=C4)=C4N=C3)=O)=O)C1	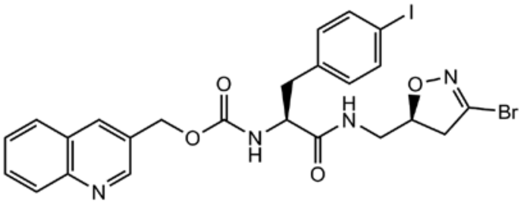	This work
43	**D83**	BrC1=NO[C@H](CNC([C@H](CC2=CC=CC=C2)N(C)C(OCC3=CC(C=CC=C4)=C4N=C3)=O)=O)C1	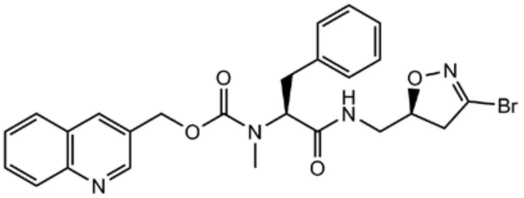	‡

44	**D84**	BrC1=NO[C@H](CNC([C@H](CC2=CC=C(C3=CC=CC=C3)C=C2)NC(OCC4=CC(C=CC=C5)=C5N=C4)=O)=O)C1	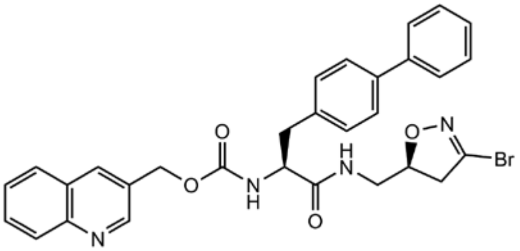	‡
45	**D85**	BrC1=NO[C@H](CNC([C@H](CCC2=CC=CC=C2)NC(OCC3=CC(C=CC=C4)=C4N=C3)=O)=O)C1	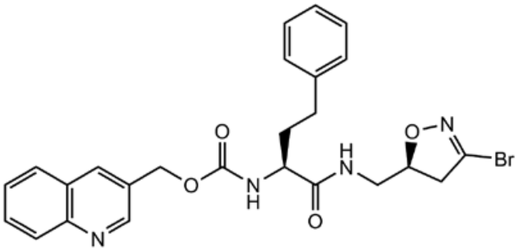	‡
46	**D86**	BrC1=NO[C@H](CNC([C@H](CC2=CC=CC3=C2C=CC=C3)NC(OCC4=CC(C=CC=C5)=C5N=C4)=O)=O)C1	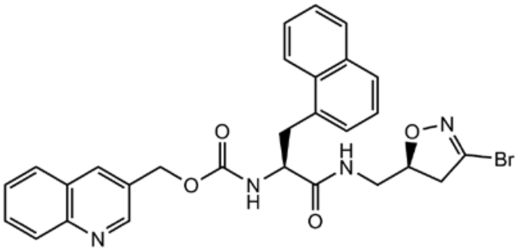	‡
47	**D87**	BrC1=NO[C@H](CNC([C@H](CC2=CC=C(O)C=C2)NC(OCC3=CC(C=CC=C4)=C4N=C3)=O)=O)C1	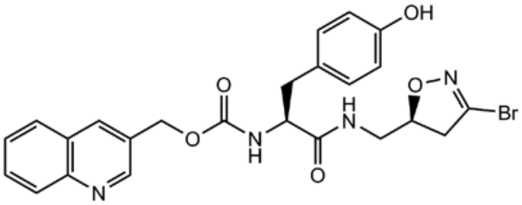	[4]
48	**D88**	BrC1=NO[C@H](CNC([C@H](CC2=CC=CC=C2)NC(OCC3=CC(C=CC=C4)=C4N=C3)=O)=O)C1	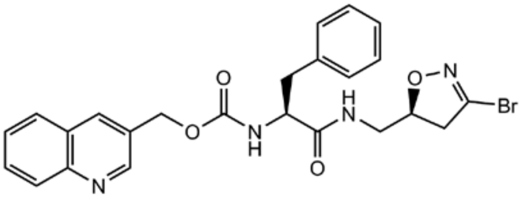	This work
49	**D91**	BrC1=NO[C@H](CNC([C@@](CC2=CC=CC=C2)(C)NC(OCC3=CC(C=CC=C4)=C4N=C3)=O)=O)C1	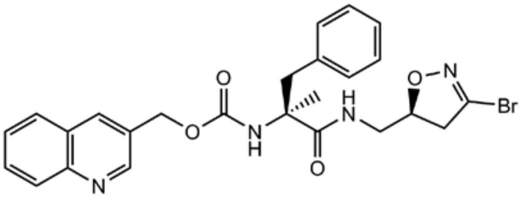	‡
50	**D92**	O=C(N1CC2=C(C=CC=C2)C[C@H]1C(NC[C@@H]3CC(Br)=NO3)=O)OCC4=CC(C=CC=C5)=C5N=C4	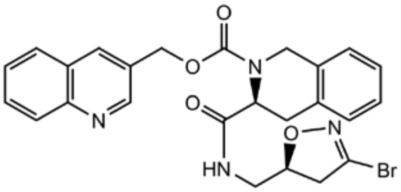	[6]

51	**D93**	BrC1=NO[C@H](CNC([C@H](CC2=CSC3C2C=CC=C3)NC(OCC4=CC(C=CC=C5)=C5N=C4)=O)=O)C1	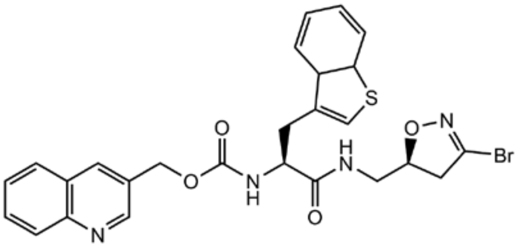	This work
52	**D94**	BrC1=NO[C@H](CNC([C@@]([C@@H](C)C2=CC=CC=C2)([H])NC(OCC3=CC(C=CC=C4)=C4N=C3)=O)=O)C1	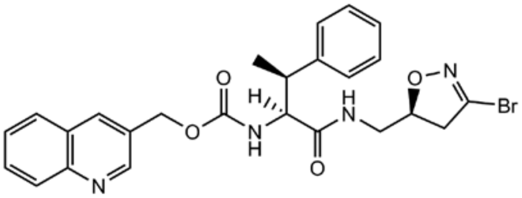	‡
53	**D95**	BrC1=NO[C@H](CNC([C@H](CC2=C(F)C=CC=C2)NC(OCC3=CC(C=CC=C4)=C4N=C3)=O)=O)C1	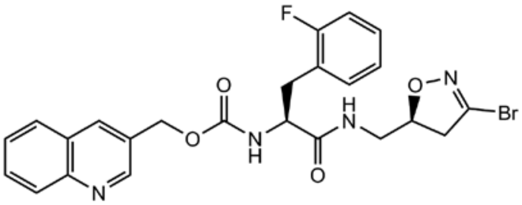	This work
54	**D96**	BrC1=NO[C@H](CNC([C@H](CC2=CC=C(F)C=C2)NC(OCC3=CC(C=CC=C4)=C4N=C3)=O)=O)C1	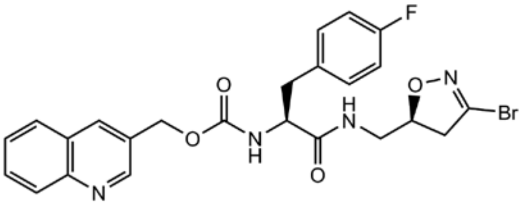	This work
55	**D97**	O=C(NC[C@H]1ON=C(C1)Br)[C@@H]2CC(C=CC=C3)=C3CN2C(OCC4=CC=C(C#C)C=C4)=O	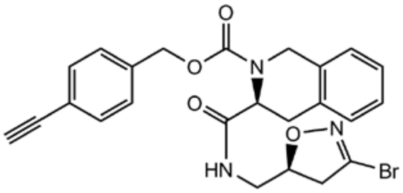	‡
56	**D98**	O=C(N1CC2=C(C(C=CC=C3)=C3N2)C[C@H]1C(NC[C@@H]4CC(Br)=NO4)=O)OCC5=CC(C=CC=C6)=C6N=C5	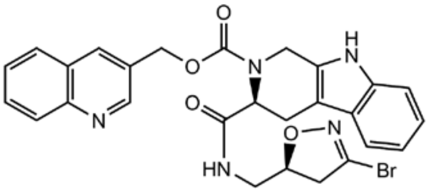	[6], ‡
57	**D101**	BrC1=NO[C@H](CNC([C@H]2N(CCC2)C(OC(C)(C)C)=O)=O)C1	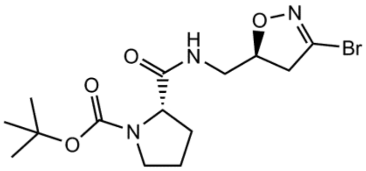	‡
58	**D102**	BrC1=NO[C@H](CNC([C@@H]2N(CCC2)C(OC(C)(C)C)=O)=O)C1	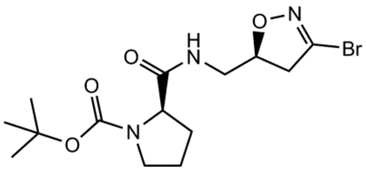	‡

59	**D103**	BrC1=NO[C@H](CNC([C@H](CC2=CNC3C2C=C(F)C=C3)NC(OC(C)(C)C)=O)=O)C1	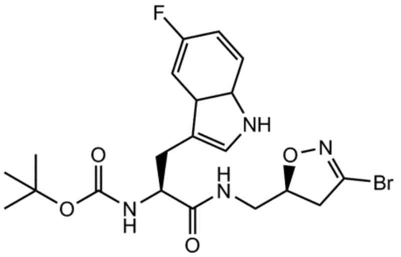	This work
60	**D104**	BrC1=NO[C@H](CNC([C@H]2N(CCC2)C(OCC3=NC(C=CC=C4)=C4N=C3)=O)=O)C1	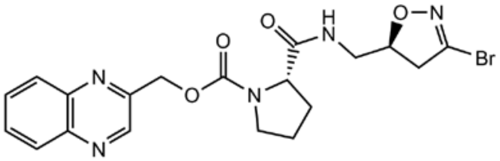	‡
61	**D105**	BrC1=NO[C@H](CNC([C@H](CC2=CNC3C2C=C(F)C=C3)NC(OCC4=NC(C=CC=C5)=C5N=C4)=O)=O)C1	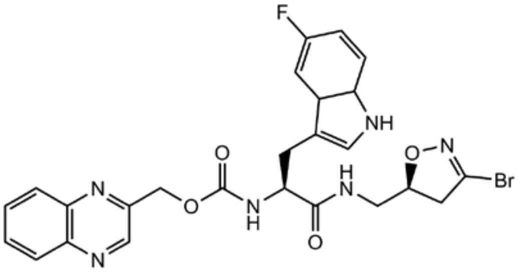	This work
62	**D106**	BrC1=NO[C@H](CNC([C@H]2N(CCC2)C(OCC3=C(C=CC=C4)C4=NC=C3)=O)=O)C1	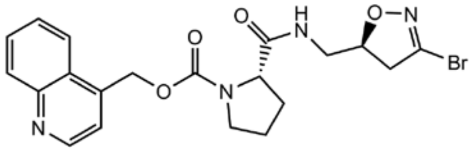	‡
63	**D107**	BrC1=NO[C@H](CNC([C@H](CC2=CNC3C2C=C(F)C=C3)NC(OCC4=C(C=CC=C5)C5=NC=C4)=O)=O)C1	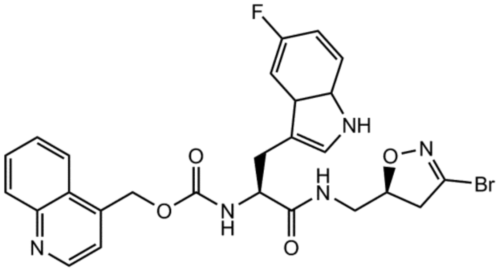	This work
64	**D108**	O=C(N1CC2=C(C(C=CC=C3)=C3N2)C[C@H]1C(NC[C@@H]4CC(Br)=NO4)=O)OCC5=NC(C=CC=C6)=C6N=C5	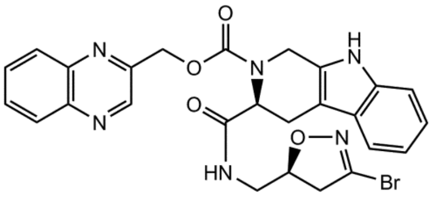	‡
65	**D111**	BrC1=NO[C@H](CNC([C@H]2N(CCC2)C(C3=NC=CN=C3)=O)=O)C1	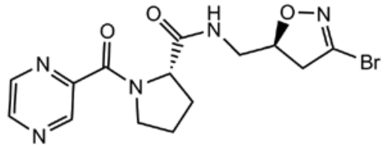	‡
66	**D112**	BrC1=NO[C@H](CNC([C@H]2N(CCC2)C(C3=NC(C=CC=C4)=C4N=C3)=O)=O)C1	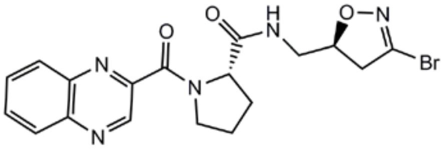	‡

67	**D113**	BrC1=NO[C@H](CNC([C@H]2N(CCC2)C(OCC3=CC=C(C#C)C=C3)=O)=O)C1	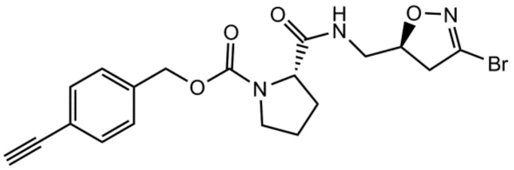	[5]
68	**D114**	ClC1=NO[C@H](CNC([C@H]2N(CC[C@@H]2O)C(OCC3=CC(C=CC=C4)=C4N=C3)=O)=O)C1	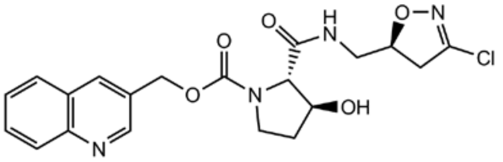	‡
69	**D115**	ClC1=NO[C@H](CNC([C@H]2N(C[C@H](O)C2)C(OCC3=CC(C=CC=C4)=C4N=C3)=O)=O)C1	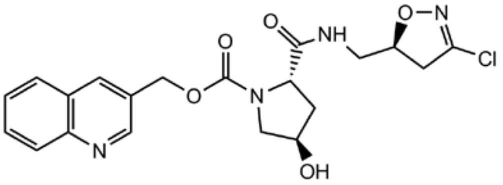	‡
70	**D116**	ClC1=NO[C@H](CNC([C@H]2N(C[C@@H](O)C2)C(OCC3=CC(C=CC=C4)=C4N=C3)=O)=O)C1	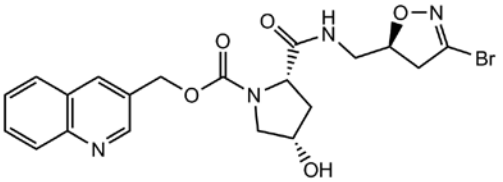	‡
71	**D117**	ClC1=NO[C@H](CNC([C@H]2N(CC[C@H]2O)C(OCC3=CC(C=CC=C4)=C4N=C3)=O)=O)C1	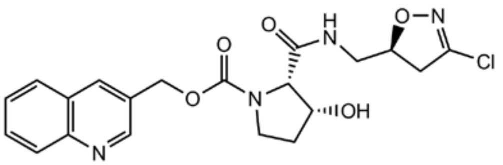	‡
72	**D121**	ClC1=NO[C@H](CNC([C@H]2N(CC2)C(OCC3=CC(C=CC=C4)=C4N=C3)=O)=O)C1	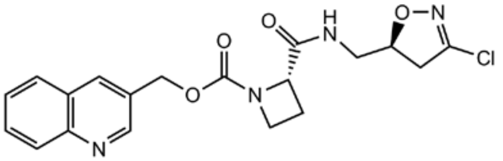	‡
73	**D131**	BrC1=NO[C@H](CNC([C@H]2N(C[C@H](OCC3=CC=CC=C3)C2)C(OCC4=CC(C=CC=C5)=C5N=C4)=O)=O)C1	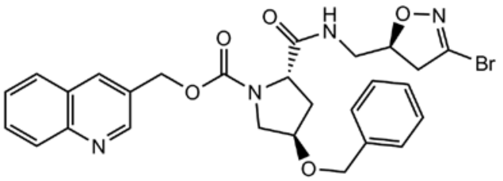	[6, 7]
74	**D132**	BrC1=NO[C@H](CNC([C@H](CC2=CNC3C2C=CC=C3)NC(OCC4=CC(C=CC=C5)=C5N=C4)=O)=O)C1	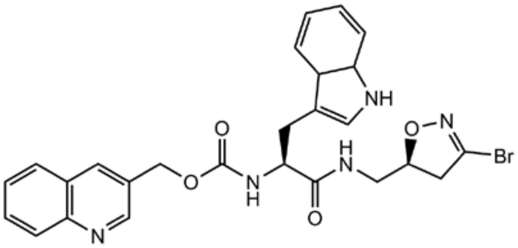	[5]
75	**D133**	O=C(N1CCCC[C@H]1C(NC[C@@H]2CC(Br)=NO2)=O)OCC3=CC(C=CC=C4)=C4N=C3	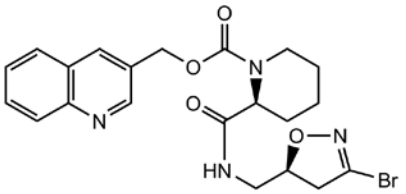	[6], ‡

76	**D134**	BrC1=NO[C@H](CNC([C@H]2N(C[C@H](O)C2)C(OCC3=CC(C=CC=C4)=C4N=C3)=O)=O)C1	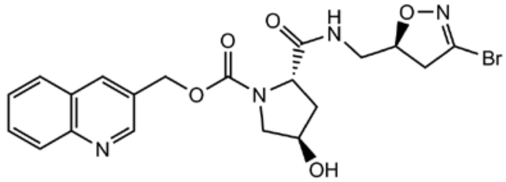	[6], ‡
77	**D135**	BrC1=NO[C@H](CNC([C@H]2N(C[C@H](OC)C2)C(OCC3=CC(C=CC=C4)=C4N=C3)=O)=O)C1	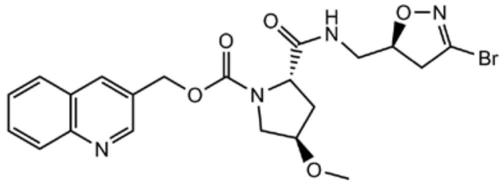	[6], ‡
78	**D136**	BrC1=NO[C@H](CNC([C@H]2N(C[C@H](OCC#C)C2)C(OCC3=CC(C=CC=C4)=C4N=C3)=O)=O)C1	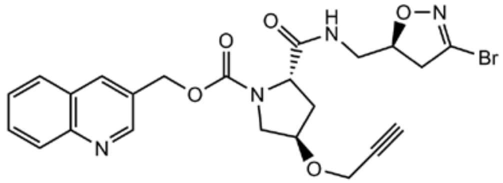	[6, 7]
79	**D137**	BrC1=NO[C@H](CNC([C@H]2N(C[C@@H](F)C2)C(OCC3=CC(C=CC=C4)=C4N=C3)=O)=O)C1	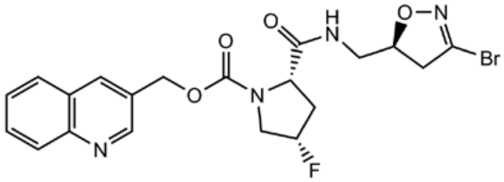	[6, 7]
80	**D138**	BrC1=NO[C@H](CNC([C@H]2N(C[C@H](F)C2)C(OCC3=CC(C=CC=C4)=C4N=C3)=O)=O)C1	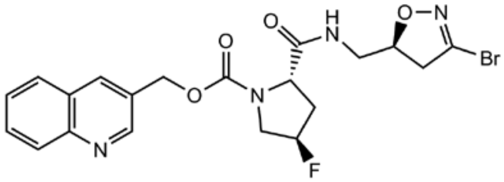	[6, 7]
81	**D141**	BrC1=NO[C@H](CNC([C@H](CC2=CNC3C2C=C(O)C=C3)NC(OCC4=CC(C=CC=C5)=C5N=C4)=O)=O)C1	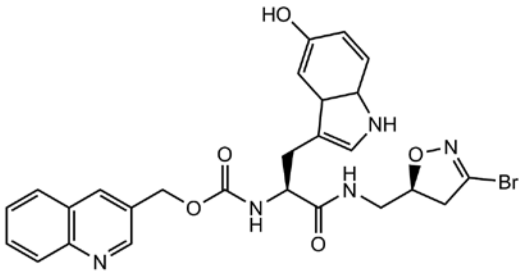	[5]
82	**D142**	BrC1=NO[C@H](CNC([C@H](CC2=CC=C(O)C=C2)NC(OCC3=CC=C(C#C)C=C3)=O)=O)C1	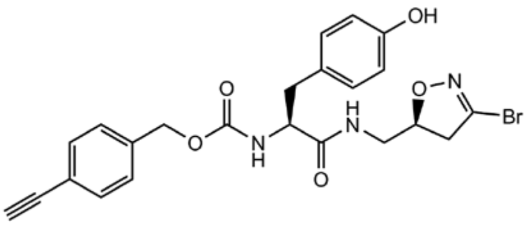	[5]
83	**D143**	BrC1=NO[C@H](CNC([C@H](CC2=CC=C(O)C=C2)NC(OCC3=CC=C(N=[N+]=[N-])C=C3)=O)=O)C1	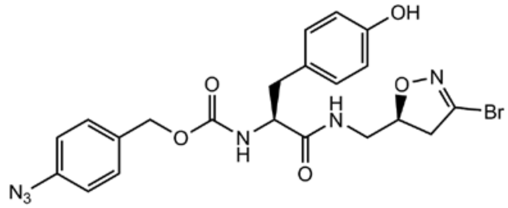	[5]

84	**I11**	O=C1NC2=CC(F)=CC=C2/C1=C\C(C)=O	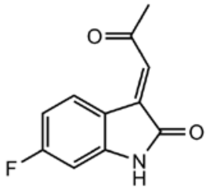	[8]
85	**I12**	O=C1NC2=CC=CC(Cl)=C2/C1=C\C(C)=O	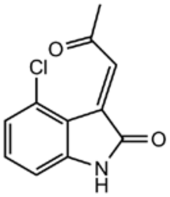	[8]
86	**I13**	O=C1NC2=CC=CC(Cl)=C2/C1=C\C(C3=CN=CC=C3)=O	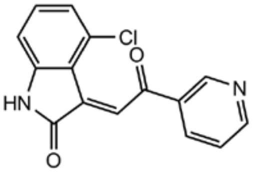	[8]
87	**I14**	O=C1NC2=CC=C(Cl)C=C2/C1=C\C(C3=CN=CC=C3)=O	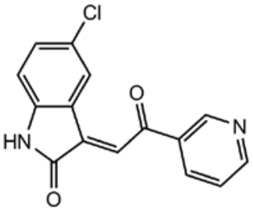	[8]
88	**I15**	O=C1NC2=CC(Cl)=CC=C2/C1=C\C(C3=CN=CC=C3)=O	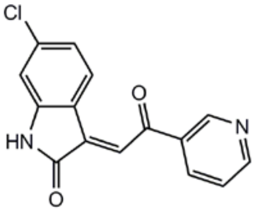	[8]
89	**I16**	O=C1NC2=C(Cl)C=CC=C2/C1=C\C(C3=CN=CC=C3)=O	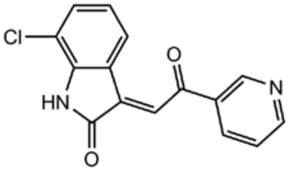	[8]
90	**I17**	O=C1N(CC2=CC=CC=C2)C3=CC=CC(Cl)=C3/C1=C\C(C4=CN=CC=C4)=O	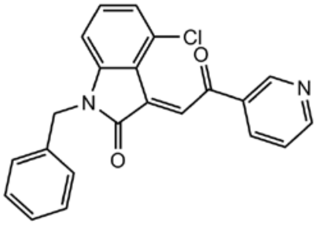	[8]
91	**I18**	O=C1NC2=CC=CC(Cl)=C2/C1=C\C(C3=CC=C(Cl)C=C3)=O	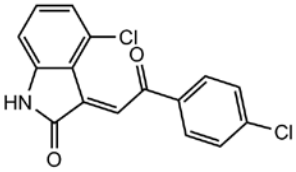	[8]

92	**M11**	O=C1N(C2=CC=CC=C2)C(SCC(NN)=O)=N C3=C1C(C4=CC=CC=C4)=CS3	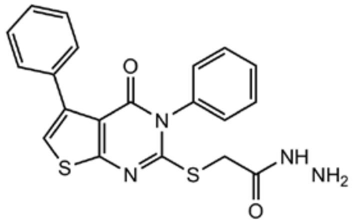	[9]
