# Supplementary material for: Dihydroisoxazole inhibitors of Anopheles gambiae seminal transglutaminase AgTG3
Source: Malar J. 2014 Jun 2;13:210. doi: 10.1186/1475-2875-13-210 (PMC4113009; doi:10.1186/1475-2875-13-210)
Supplement: Additional file 3: Table S2 — AgTG3 IC50 for 27 dihydroisoxazole inhibitors. [file 1475-2875-13-210-S3.pdf]

Supplementary Table 2. AgTG3 IC_{50} for 27 dihydroisoxazole inhibitors

	Structure	IC_{50}		Structure	IC_{50}
D56	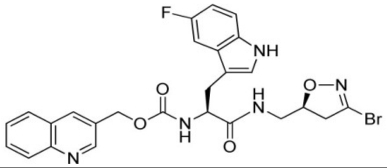	3.1	D66	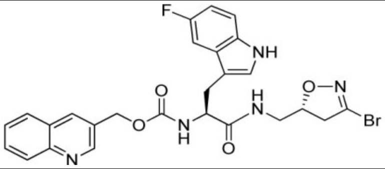	>100
D61<br>D87	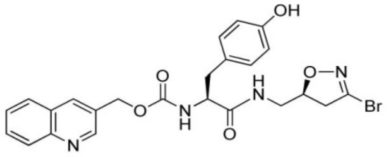	65	D62	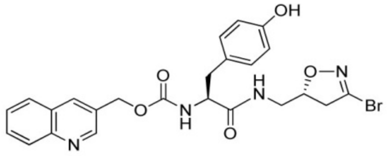	>100
D27	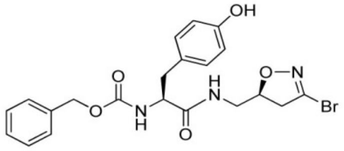	>100	D26	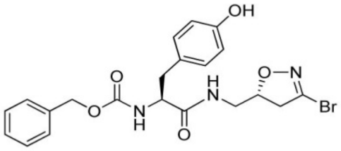	>100
D73	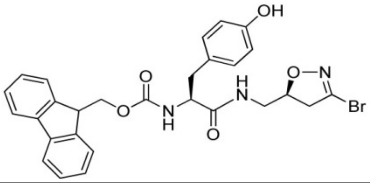	>100	D71	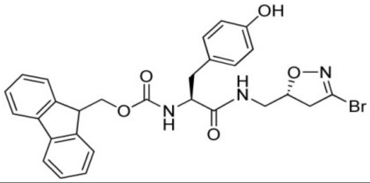	>100
D88	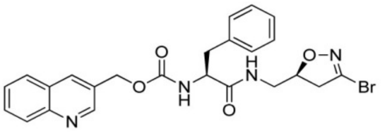	29	D81	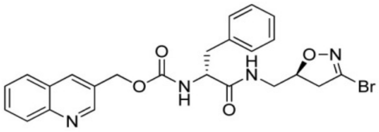	>100
D82	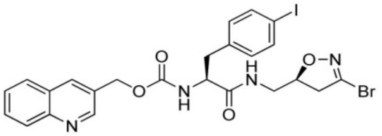	>100	D83	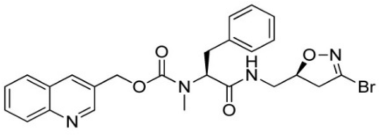	>100
D84	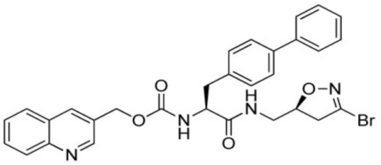	>100	D85	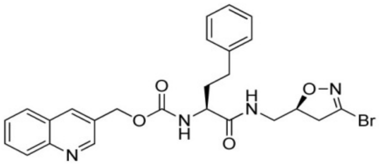	>100
D86	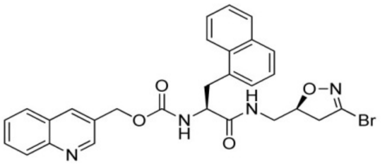	>100	D91	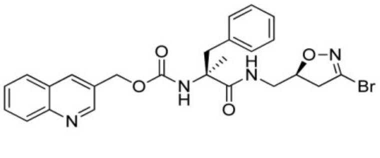	>100
D93	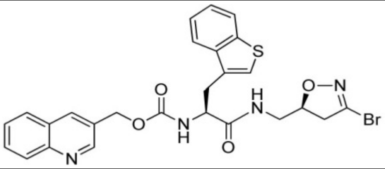	18	D94	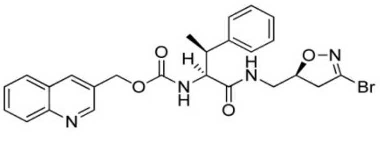	84
D95	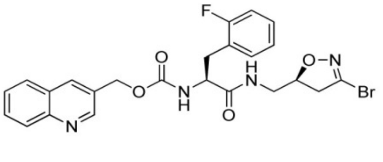	28	D96	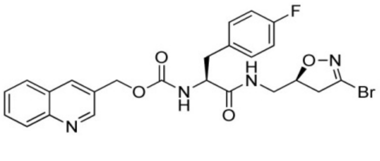	66

Structure	IC50	Structure	IC50
D105 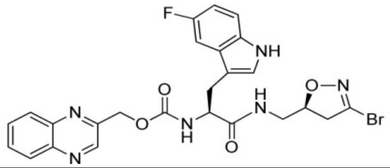	9.7	D103 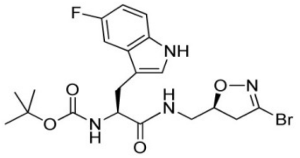	56
D132 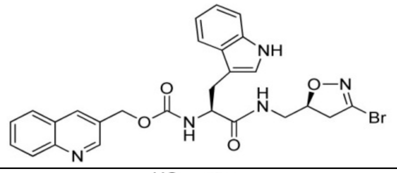	6.1	D107 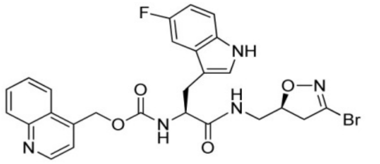	12.2
D141 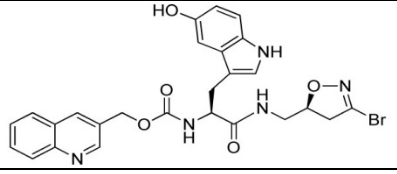	4.3		
D142 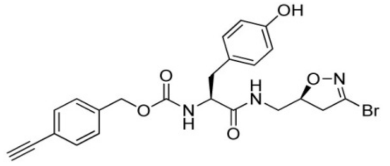	>100	D143 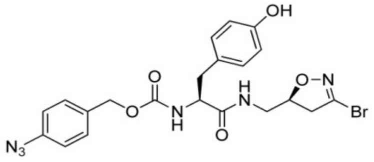	>100
