# Supplementary material for: Dihydroisoxazole inhibitors of Anopheles gambiae seminal transglutaminase AgTG3
Source: Malar J. 2014 Jun 2;13:210. doi: 10.1186/1475-2875-13-210 (PMC4113009; doi:10.1186/1475-2875-13-210)
Supplement: Additional file 4: Table S3 — Graphical comparison of AgTG3 IC50 for 27 dihydroisoxazole inhibitors. [file 1475-2875-13-210-S4.pdf]

Supplementary Table 3. Graphical comparison of AgTG3 IC_{50} for 27 dihydroisoxazole inhibitors

Structure	Dose-response	Structure	Dose-response
D56 (IC_{50} 3.1 μM)<br>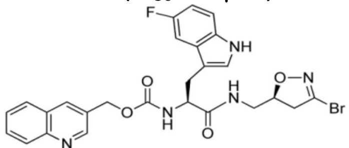<br>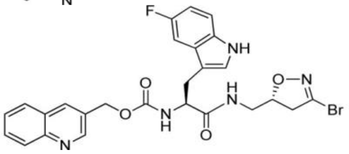<br>D66 (IC_{50} >100 μM)	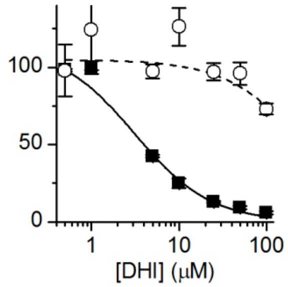	D61 (IC_{50} 65 μM)<br>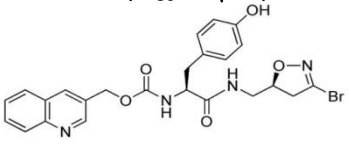<br>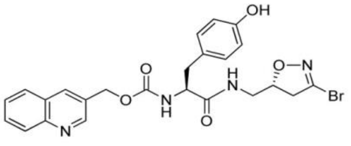<br>D62 (IC_{50} >100 μM)	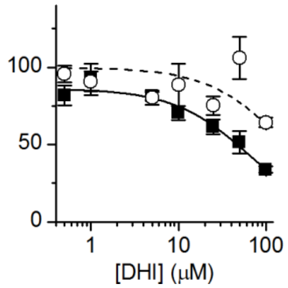
D27 (IC_{50} >100 μM)<br>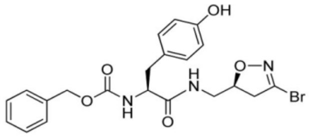<br>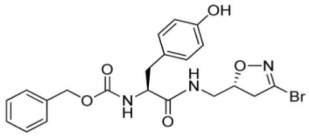<br>D26 (IC_{50} >100 μM)	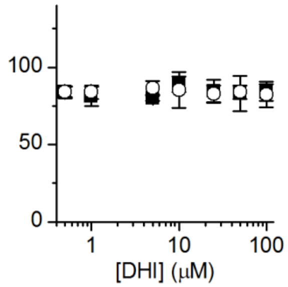	D73 (IC_{50} >100 μM)<br>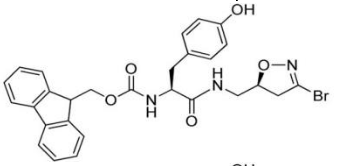<br>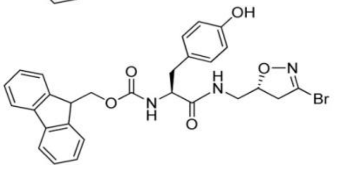<br>D71 (IC_{50} >100 μM)	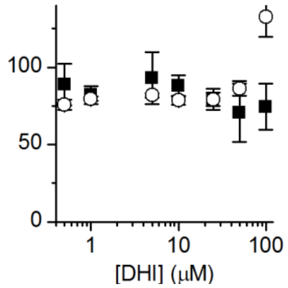
D88 (IC_{50} 29 μM)<br>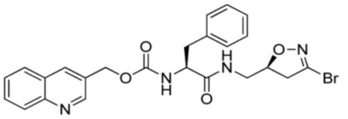<br>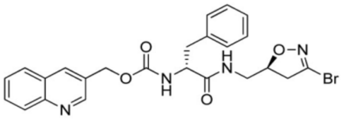<br>D81 (IC_{50} >100 μM)	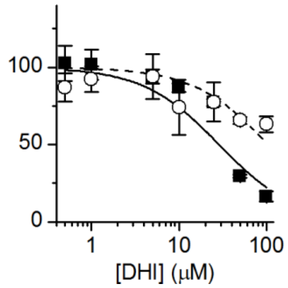	D82 (IC_{50} >100 μM)<br>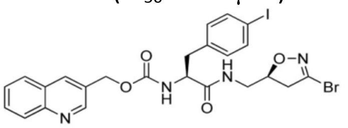<br>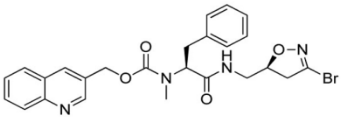<br>D83 (IC_{50} >100 μM)	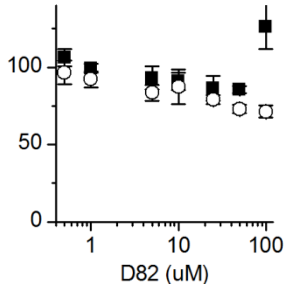
D84 (IC_{50} >100 μM)<br>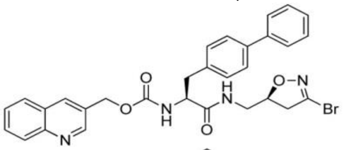<br>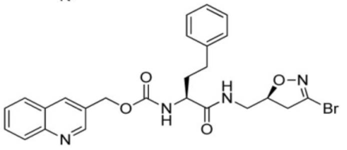<br>D85 (IC_{50} >100 μM)	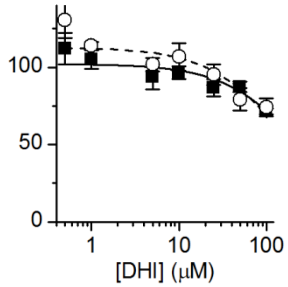	D86 (IC_{50} >100 μM)<br>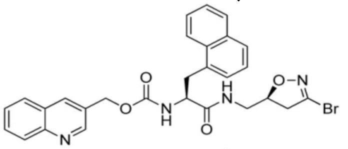<br>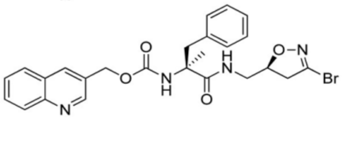<br>D91 (IC_{50} >100 μM)	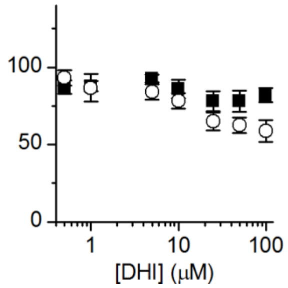

**Structure**	**Dose-response**	**Structure**	**Dose-response**
D93 (IC_{50} 18 μM)<br>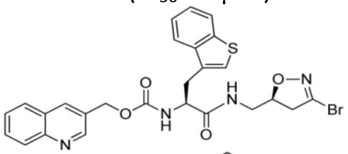<br>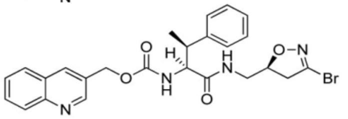<br>D94 (IC_{50} 84 μM)	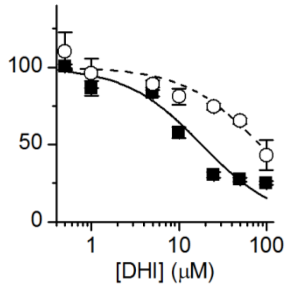	D95 (IC_{50} 28 μM)<br>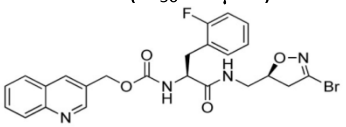<br>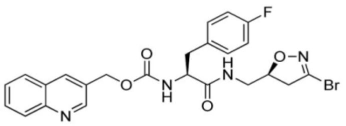<br>D96 (IC_{50} 66 μM)	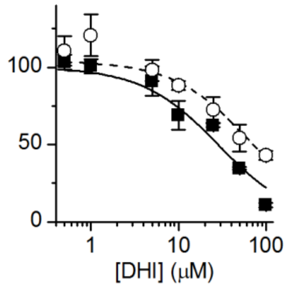
D105 (IC_{50} 9.7 μM)<br>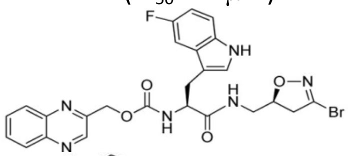<br>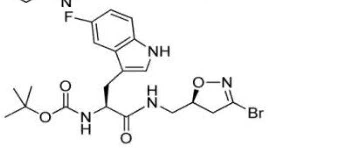<br>D103 (IC_{50} 56 μM)	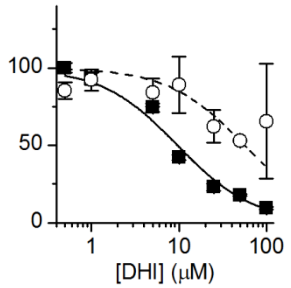	D132 (IC_{50} 6.1 μM)<br>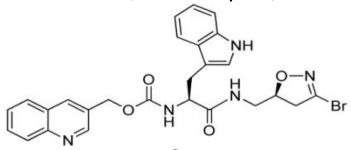<br>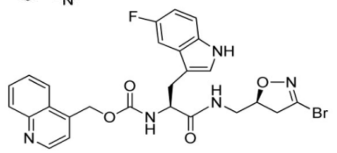<br>D107 (IC_{50} 12 μM)	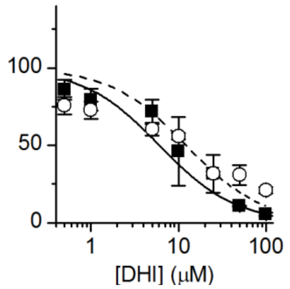
D141 (IC_{50} 4.3 μM)<br>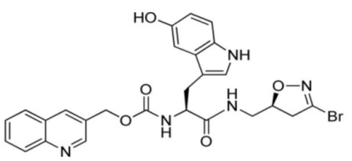	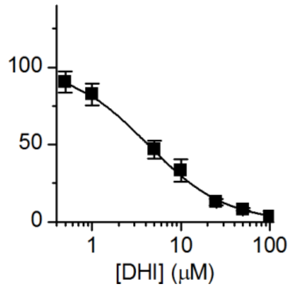	D142 (IC_{50} >100 μM)<br>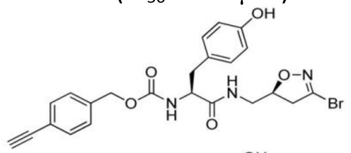<br>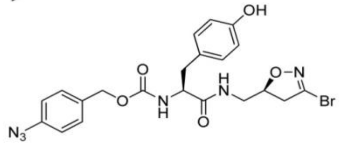<br>D143 (IC_{50} >100 μM)	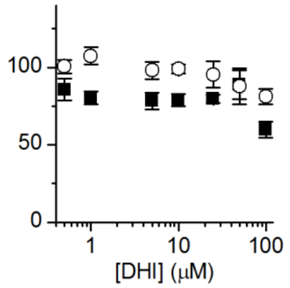
